# A Fully Biodegradable Ferroelectric Skin Sensor from Edible Porcine Skin Gelatine

**DOI:** 10.1002/advs.202005010

**Published:** 2021-05-07

**Authors:** Sujoy Kumar Ghosh, Jonghwa Park, Sangyun Na, Minsoo P. Kim, Hyunhyub Ko

**Affiliations:** ^1^ School of Energy and Chemical Engineering Department of Energy Engineering Ulsan National Institute of Science and Technology (UNIST) Ulsan Metropolitan City 44919 Republic of Korea

**Keywords:** biodegradable, electronic skin, ferroelectric, gelatine, nanogenerators, sensors

## Abstract

High‐performance biodegradable electronic devices are being investigated to address the global electronic waste problem. In this work, a fully biodegradable ferroelectric nanogenerator‐driven skin sensor with ultrasensitive bimodal sensing capability based on edible porcine skin gelatine is demonstrated. The microstructure and molecular engineering of gelatine induces polarization confinement that gives rise the ferroelectric properties, resulting in a piezoelectric coefficient (*d*_33_) of ≈24 pC N^−1^ and pyroelectric coefficient of ≈13 µC m^−2^K^−1^, which are 6 and 11.8 times higher, respectively, than those of the conventional planar gelatine. The ferroelectric gelatine skin sensor has exceptionally high pressure sensitivity (≈41 mV Pa^−1^) and the lowest detection limit of pressure (≈0.005 Pa) and temperature (≈0.04 K) ever reported for ferroelectric sensors. In proof‐of‐concept tests, this device is able to sense the spatially resolved pressure, temperature, and surface texture of an unknown object, demonstrating potential for robotic skins and wearable electronics with zero waste footprint.

## Graphical abstract

Fully transient ferroelectric electronic skin (e‐skin) is demonstrated based on edible porcine skin gelatine. The interlocked molecular engineering induces ferroelectricity in gelatine. The e‐skin detects and discriminates the lowest possible resolution of pressure and temperature ever reported for ferroelectric sensors. In proof‐of‐concept tests, the potential applications towards robotic skins and wearable electronics are demonstrated with zero waste footprint.

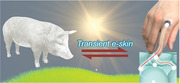

## 1 Introduction

Materials with ferroelectricity, a subset of piezo/pyroelectricity, enable various applications including but not limited to non‐volatile memory devices, temperature and tactile sensors, transducers, actuators, and energy harvesters.^[^ ^1^, ^2^ ^]^ However, traditional inorganic and organic ferroelectric materials rarely meet the requirements of next‐generation electronics considering the biocompatibility, biodegradability, or at least recyclability. Hence, the development of eco‐friendly and sustainable technologies is required.^[^ ^1^, ^2^, ^3^ ^]^ Most existing electronic devices are non‐decomposable, leading to the generation of large volumes of electronic waste (e‐waste) (53.6 million tonnes in 2019 and an expected 74.7 million tonnes by 2030 worldwide), which is threatening the health of ecological systems.^[^ ^4^ ^]^ Since e‐waste contains hazardous components, even the recycling of these materials contaminates ecosystems with toxins that can enter living bodies by inhalation, skin exposure, and oral intake of contaminated foods.^[^ ^5^ ^]^ Hence, transient and ingestible electronics made from completely biodegradable and edible materials are particularly attractive. Such devices can be completely degraded by decomposition and digestion processes after a certain period of use, thus reducing the amount of e‐waste produced.^[^ ^6^ ^]^ Over the last decade, a range of biodegradable and bioresorbable devices were demonstrated as in vivo sensors and stimulators based on triboelectric effect which shown a new direction toward transient implantable technology.^[^ ^7^, ^8^, ^9^ ^]^ One potential use of transient electronics is in flexible electronic skin (e‐skin) sensors for wearable devices and robotic skin applications, where ultrasensitive temperature, pressure, and texture perception capabilities are required to monitor the body conditions and surrounding environment.^[^ ^10^ ^]^ In the pursuit of this goal, the combination of pyro/piezoelectricity is required for highly sensitive, multimodal, and self‐powered functionalities.^[^ ^11^ ^]^ However, most previously developed multimodal e‐skins are based on non‐decomposable organic/inorganic materials, which continue to contribute to the global e‐waste problems. The development of a biodegradable and edible e‐skin with highly sensitive and multimodal sensing capabilities remains challenging.^[^ ^12^, ^13^ ^]^

Gelatine is a natural biological material with intrinsic biodegradability, low temperature processability, low cost (compared with other biodegradable polymers; Table S1, Supporting Information), and flexibility. Hence, it is considered as the most promising candidate for the development of biodegradable and eco‐friendly transient electronics systems.^[^ ^14^, ^15^ ^]^ Gelatine has been extensively used as an edible material in drug delivery systems^[^ ^16^ ^]^ and recently in electronics and soft robotics.^[^ ^17^ ^]^ The main challenge of gelatine for e‐skin applications lies in its low pyro/piezoelectric coefficients and instability in humid environments.^[^ ^18^ ^]^ In this scenario, nature‐inspired engineering design can enhance the functionality of gelatine for high‐performance e‐skins. Of particular interest, an important feature of biological skin is the interlocked micro‐ridge structure of epidermal‐dermal layers. This interlocked structure plays a key role in the precise perception of pressure and temperature through the localization and amplification of stress and temperature within the intermediate ridge tips and their effective transmission to the underlying mechanoreceptors in the dermis layer.^[^ ^19^ ^]^ Imitating the structure and functionality of human skin, herein, a fully biodegradable gelatine‐based material with high ferroelectric properties is demonstrated using the physical confinement of molecular structures of gelatine within interlocked microstructures. The ferroelectric gelatine e‐skin is self‐powered and highly sensitive to both temperature and pressure stimuli due to its large pyro/piezoelectric coefficients, respectively, and finally biodegraded with zero waste footprint.

## 2 Results

### 2.1 Fabrication of a Biodegradable Ferroelectric Sensor from Porcine Skin Gelatine

**Figure 1:**
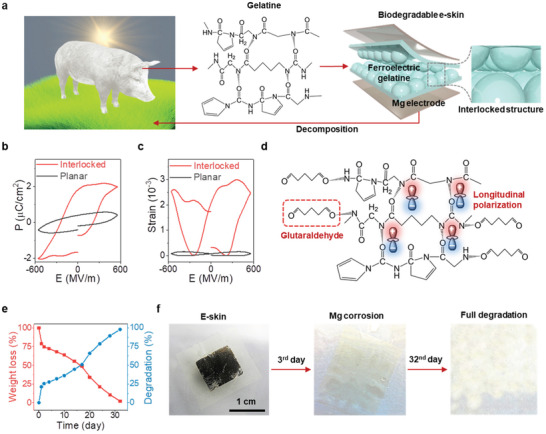
Biodegradable ferroelectric gelatine e‐skin. a) Schematic of the concept of gelatine‐based transient “green” electronics from biosynthesis to decomposition. b) P–E hysteresis loop. c) Strain vs electric field hysteresis loop. d) Charge imbalance mechanism during cross‐linking by glutaraldehyde. e) Complete biodegradability demonstrated using an in vitro biodegradation test showing day‐by‐day weight loss, degradation rate, and f) corresponding photographs of the e‐skin degrading in the PBS solution.

**Figure** **1**a shows an ideal recycling process for decomposable transient electronics with zero waste footprint, which consists of water‐based biosynthesis of gelatine extracted from porcine skin, followed by device application, and finally full decomposition in the environment. No harmful solvents or chemicals are involved in any part of the process. For the e‐skin fabrication, Mg was used as the metal for the electrodes as it is biodegradable and biocompatible. Mg is commonly used in biodegradable stents and is considered as an essential nutrient.^[^ ^20^ ^]^ The as‐manufactured lightweight (2.75 mg cm^−2^; Figure S1a,b, Supporting Information) and thin (42.2 µm; Figure S1c, Supporting Information) gelatine nanogenerator‐based e‐skin can be conformably attached on the curvilinear surface of the human body (Figure S1d, Supporting Information). For the physically confined molecular structures to induce the polarization confinement and enhance the ferroelectric properties of gelatine, an interlocked gelatine microstructure was fabricated by arranging microstructured (e.g., microdome, micropyramid, and micropillar) surfaces facing each other in interlocked structures (Figure S2, Supporting Information). All of the microstructures had similar feature sizes (8 µm diameter, 15 µm pitch, and 4 µm height).

For the characterization of the ferroelectricity of the gelatine films, the polarization (*P*) level of the interlocked gelatine was investigated using the electric field (*E*)‐induced hysteresis loop. While the interlocked gelatine microdome film exhibited a good switchable polarization loop, a lossy hysteresis loop was observed for the planar gelatine film (Figure 1b). Additionally, the interlocked microdome film displayed a switchable peak in the current density ($J\;=\frac{dP(t)}{dt}$) hysteresis loop *(J–E*) due to the dipole reversal under an electric field, which was not observed for the planar gelatine (Figure S3, Supporting Information). As a result, the interlocked microdome film had a remnant polarization (*P*_r_ ≈ 1.8 µC cm^−2^) that was 4.5 times higher than that of planar gelatine film (≈0.4 µC cm^−2^). Considering that the longitudinal piezoelectric coefficient $d_{33}=\;-\frac{P_{r}}{Y}$, where *Y* is the Young's modulus of the gelatin film (Figure S4, Supporting Information),^[^ ^21^ ^]^ the interlocked gelatine film had a *d*_33_ value of *≈*24 pC N^−1^, which is 6 times higher than that of the planar gelatine film (*d*_33_ *≈* 4 pC N^−1^). The strain hysteresis loop was dependent on the electric field and had a butterfly shape (Figure 1c), which implies the converse effect of piezoelectricity. The electrostriction coefficient was evaluated using the equation of electrostriction, *d*_33_ =  2*Qε*_r_ *ε*_0_ *P*_r_, where *ε*_r_  ≈ 12 is the relative permittivity of gelatin.^[^ ^21^, ^22^ ^]^ Evidently, the piezoelectricity‐induced strain (≈3 × 10^−3^) in the interlocked structure was higher than the electrostriction‐induced strain (≈0.16 × 10^−3^) in the planar film. As a result, the interlocked structure had an electrostriction coefficient (*Q* ≈ 6.3 m^4^ C^−2^) that was 1.3 times higher than that of the planar gelatine (*Q* ≈ 4.7 m^4^ C^−2^).

The highly enhanced ferroelectricity of the gelatine microdome film is attributed to the enhanced molecular packing by the spatial confinement within the microdome structure. The interlocked gelatine microdome film had a higher crystallinity (29%) than that (15%) of the planar one (Figure S5a,b, Supporting Information). Interestingly, a shift in the crystalline peak toward higher angles was observed in the X‐ray diffraction pattern (XRD) pattern; for example, 2*θ* ≈ 7° for the planar film to 7.64° for the microdome film (Figure S5a,c, Supporting Information). This shift indicates that the microdome film had a higher intermolecular packing structure with a lower spacing (*d*) of 1.15 nm than that of planar (*d* ≈ 1.26 nm) and other microstructured films (*d* ≈ 1.24 and 1.20 nm for micropillar and micropyramid, respectively). This enhanced packing within the microdome structure is attributed to the higher vorticity of the spinning gelatine solution within the reverse microdome structure compared to those of other structures during the film fabrication by spin coating (finite element method (FEM) simulation in Figure S6, Supporting Information). The FEM simulation also showed that the viscous stress on the spinning solution is the highest within the reverse microdome structure (Figure S7, Supporting Information), which indicates the largest stretching and confinement of polymer chains within the microdome structure. Additionally, the viscous stress is directed along the height direction of the microstructures, leading to the stretching and confinement of polypeptide chains. The shifting and absorption ratio of stretching vibrational bands of the amide group in Fourier transform infrared spectroscopy (FT‐IR) spectra (Figure S8, Note S1, Supporting Information) evidences the larger stretching and spatial confinement of oriented polypeptide chains in the microdome compared to the other structures. In general, collagenous polypeptide chains show shear piezoelectricity by N (—NH_2_) and C (—COOH) terminal telopeptides forming a quasi‐hexagonal symmetry (C_6_).^[^ ^23^ ^]^ The decreased intermolecular d‐spacing in the microdome structure enhances the intermolecular van der Waals interaction between polypeptide chains, resulting in the larger anisotropic collagen structure.^[^ ^24^ ^]^ Further introduction of anisotropy through cross‐linking by glutaraldehyde (GA), in which, side amino acid groups become inactive (Figure 1d), may lower the symmetry compared to the quasi‐hexagonal symmetry (C_6_), providing the gelatine microdome with high ferroelectricity. This behavior was also observed in a previous theoretical study based on a DFT quantum mechanical simulation, where the addition of —OH groups significantly lowered the crystal symmetry and enhanced the polarization with the piezoelectric coefficient up to 28 pC N^−1^ for hydroxy‐L‐proline which is one of the major building block of gelatine.^[^ ^25^ ^]^ This type of ferroelectricity induced by symmetry lowering (from D4 to C4 symmetry) was also observed in lysozyme protein during the crystal growth process.^[^ ^26^ ^]^ In contrast, the planar gelatine film without the physical confinement of polypeptide chains only produced vertical piezoelectricity, but not the ferroelectricity.

Additionally, the cross‐linking of gelatine by GA increased the moisture stability, evidenced by a lower degree of swelling and hydrophobicity of the gelatine (Figure S9, Supporting Information), without compromising optical transparency (Figure S10, Supporting Information). As a result, while the cross‐linked planar gelatine e‐skin nanogenerator showed a stable piezoelectric output voltage and current up to 70% relative humidity, the non‐cross‐linked one shows no stable output responses under relative humidity values of 30–80% (Figure S11, Supporting Information). Furthermore, our gelatine e‐skin nanogenerator is biodegradable and decomposes within a month, which is advantageous for transient device applications. In an in vitro decomposition study, our gelatine e‐skin nanogenerator was immersed in pH 7.4 phosphate buffered saline (PBS) solution at 37 °C to simulate the body fluid environment and agitated at 100 rpm. The percentage of weight loss and degradation rate indicated that the e‐skin nanogenerator was completely degraded over 32 days of immersion (Figure 1e). The structural integrity of our gelatine e‐skin nanogenerator persisted for 17 days, whereas, the Mg electrodes totally corroded within 3 days (Figure 1f ) due to the rapid reactions between Mg and water (Mg + 2H_2_O → Mg^2 +^ + 2OH^−^ + H_2_↑;  Mg^2 +^ + 2OH^−^↔Mg(OH)_2_↓).^[^ ^27^ ^]^ After 20 days, the integral structure of the e‐skin nanogenerator swelled due to fracturing of the polymer backbone. Then, rapid autocatalytic hydrolysis and bulk degradation occurred up to day 24 (Figure S12, Supporting Information), after which time the integral structure of the e‐skin nanogenerator ruptured and was fully decomposed by day 32 (Figure 1f).

### 2.2 Pyroelectric and Piezoelectric Property of a Microstructured Gelatine Skin Sensor

**Figure 2:**
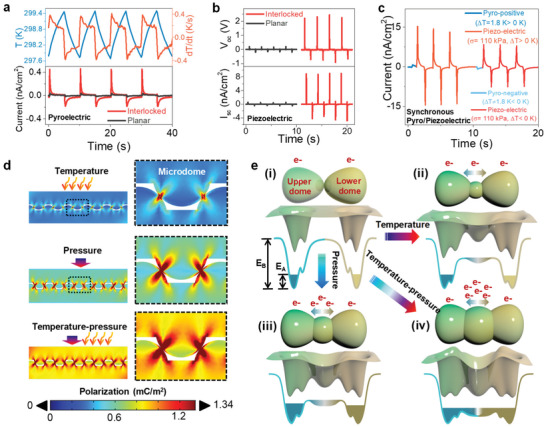
Energy harvesting performance of the ferroelectric gelatine skin sensor. a) Comparison of the pyroelectric output current of planar and interlocked devices (lower panel) under thermal fluctuation of ∆*T* ≈ 1.8 K, $\frac{dT}{dt}\approx \;0.4\;K\;s^{-1}$ (upper panel). b) The piezoelectric open‐circuit voltage (*V*_oc_) and short‐circuit current (*I*_sc_) for stress *σ* of ≈113 kPa. c) The synchronous pyro/piezoelectric output current from the interlocked e‐skin under simultaneous ∆*T* ≈ 1.8 K and *σ* ≈ 113 kPa. d) FEM‐based theoretical simulations of the polarization distribution under temperature, pressure, and concurrent temperature–pressure stimuli. e) Schematic of the 3D polarized‐electron‐cloud‐potential‐well model and 2D potential energy profile of two interfacing atoms at the interlocked microdome side: i) without a stimulus, or with ii) temperature stimulus, iii) pressure stimulus, and iii) concurrent temperature–pressure stimuli.

The ferroelectric gelatine e‐skin nanogenerator with both pyroelectric and piezoelectric behavior is able to detect temperature changes in its surroundings and responds to touch, enabling dexterous manipulation of objects. **Figure** **2**a shows that the interlocked gelatine nanogenerator generated a pyroelectric current of ≈0.46 nA cm^−2^, which is 11 times higher than that (0.04 nA cm^−2^) of the planar one in response to a temperature difference (Δ*T*) of 1.8 K and a temporal temperature change (d*T*/d*t*) of 0.4 K s^−1^. The pyroelectric output current depends on the geometry of the microstructures in the order of microdome > micropyramid > micropillar > planar structures (Figure S13, Supporting Information). Furthermore, the interlocked microdome nanogenerator exhibited higher piezoelectric outputs (open‐circuit voltage, *V*_oc_ ≈ 2.3 V and short‐circuit current, *I*_sc_ ≈ 8.9 nA cm^−2^) (Figure 2b) than the planar one (*V*_oc_ ≈ 0.2 V; *I*_sc_ ≈ 0.5 nA cm^−2^) under a stress of 113 kPa. For different microstructures, the piezoelectricity follows the same tendency of pyroelectricity (Figure S14, Supporting Information). The maximal harvested piezoelectric power output (0.3 µW/cm^2^ at an external load resistance of 8 MΩ) was sufficient to operate commercial light emitting diodes (Figure S15, Supporting Information). The piezoelectric performance of the interlocked gelatine nanogenerator was superior to those of state‐of‐the‐art biomaterial‐based nanogenerators (detailed comparison is shown in Table S2, Supporting Information), which are generally not highly biodegradable.

Owing to its excellent pyroelectric and piezoelectric functionality, the interlocked gelatine e‐skin nanogenerator is capable of simultaneous sensing of temperature and pressure under concurrent variations in temperature (Δ*T*  ≈  1.8 K) and pressure (*σ* ≈ 113 kPa) (Figure 2c). Interestingly, the pyroelectric coupling resulted in a higher piezoelectric current output (≈ 15 nA cm^−2^) during heating than during cooling (≈8 nA cm^−2^). This result indicates thermally induced strain‐coupled piezoelectricity, where the polarization change (Δ*P*) is influenced by both Δ*T* and *σ* as follows: Δ*P*  = *d_ij_* *σ* + ΠΔ*T*, where *d_ij_* and Π are the piezo‐ and pyroelectric coefficients, respectively.^[^ ^28^ ^]^ To understand the effect of the specific microstructure on the combined pyro/piezoelectricity, a theoretical FEM simulation was performed (see Note S2, Supporting Information). The interlocked nanogenerator showed a greater degree of polarization under the simultaneous temperature and pressure stimuli compared to the conditions of individual temperature or pressure stimulus (Figure 2d). Interestingly, the maximum polarization was observed at the interfacial area of the interlocked region due to the polarization amplification through the confinement of thermal strain (due to heat flow confinement) and mechanical stress at the interlocked interface (Figure S16, Supporting Information). Importantly, this strain and stress confinement was the highest for the interlocked microdome structure, followed by micropyramid > micropillar > planar structures, which is consistent with the experimental results. No such confinement was observed for the planar device. This confinement effect enhances the charge carrier density under the concurrent pyro/piezoelectricity.

To understand the effect of the interlocked microstructure on the pyro/piezoelectricity of gelatine, a polarized electron‐cloud‐potential‐well model is proposed to explain the phenomena (Figure 2e).^[^ ^29^ ^]^ Here, polarized electron clouds are formed by spatially localized electrons in hydrogen bonds present in both inter‐ and intra‐chain polypeptide bonds of gelatine. Quantum mechanically, a hydrogen bond system has an asymmetric double‐well potential (Figure 2e (i)).^[^ ^30^ ^]^ Here, *E*_A_ is the occupied energy levels of electrons present in the upper and lower microdomes and *E*_B_ is the required energy for electrons to oscillate within the interlocked region. Under external stimuli such as temperature (Figure 2e (ii)) and pressure (Figure 2e (iii)), the polarized electron clouds at the interface of the interlocked region come into close proximity within the repulsive force region in the interaction potential of the two systems, which results in mutual polarization and the development of a polarization gradient. Gelatine with the hydrogen bonding system has an ionic character within peptides.^[^ ^30^ ^]^ Therefore, upon close proximity, that is, at least in the region where atomic‐scale contact occurs under pressure and temperature changes, the interactions among ions, dipoles, and induced dipoles become prominent. In this case, the ion–ion (≈$\frac{1}{r^{2}}$, where *r* is the intermediate distance), ion–dipole ($\approx \frac{1}{r^{4}}$), and ion–induced‐dipole interactions are much stronger than dipole–dipole, dipole–induced‐dipole, and induced‐dipole–induced‐dipole interactions ($\approx \frac{1}{r^{6}}$) because the charge of any ion is much higher than that of the dipole.^[^ ^31^ ^]^ Therefore, additional electron cloud polarization is induced by the synergistic effect of these noncovalent inter‐ and intra‐molecular forces. These noncovalent forces change the charge distribution of neighboring atoms, which in turn can greatly influence the activation barriers. Therefore, overlapping of the two electron cloud systems occur. This electron cloud overlapping lowers the potential barrier between the two systems to initiate the tunneling dynamics. In addition, the polarization gradient increases the width and reduces the depth of the potential well. Therefore, electrons easily pass through the potential barriers to achieve inter‐well motion. Owing to the thermally induced non‐harmonic oscillation of the intrinsic polarized electron cloud, the temperature‐induced polarization gradient was lower than that the pressure‐induced one, as indicated by the FEM results (Figure 2d). Therefore, the thermally induced shallowing of the potential barrier (Figure 2e (ii)) was less than that caused by the pressure (Figure 2e (iii)). As a result, the output current under pyroelectricity was lower than that under piezoelectricity. However, in the case of the three potential wells and two potential barriers, electrons are likely to be trapped in one of the two outer potential wells caused by the pressure and temperature. Therefore, these trapped electrons require higher excitation energy to oscillate between electrodes. In contrast, under simultaneous temperature and pressure stimuli (Figure 2e (iv)), the generation of a large potential gradient (Figure 2d) results in similar depths of all three potential wells. Therefore, the probability of electron oscillations in any one of the three potential wells is similar and the electrons can easily jump between the wells. In general, owing to the lower potential barrier at the interface, interlocked gelatine devices have significantly higher sensitivity, under low pressure and temperature variations, than that of planar devices, where no such polarization gradient or confinement effects occur.

**Figure 3:**
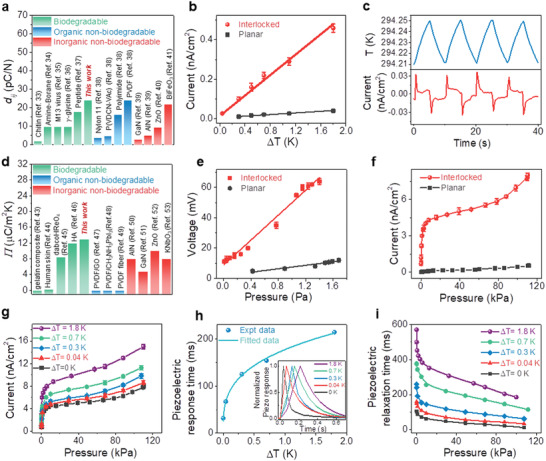
Temperature and pressure sensing performance of the gelatine skin sensor. a) Comparison of the piezoelectric strain coefficient (*d_ij_*) of the interlocked microdome gelatine with various biodegradable and non‐biodegradable (organic and inorganic) materials. b) ∆*T*‐dependent pyroelectric output current (*n* = 5). c) Time‐dependent temperature profile of ∆*T* ≈ 0.04 K (upper panel) applied to the interlocked microdome device and its corresponding pyroelectric output current (lower panel). d) Comparison of the pyroelectric coefficient (*Π*) of the interlocked microdome gelatine with various biodegradable and non‐biodegradable (organic and inorganic) materials. HA: hydroxyapatite e) Piezoelectric output voltage as a function pressure within the range of 0.005−2 Pa (*n =* 5). The Pearson's correlation coefficient, which measures the strength of linear relationship between paired data, was 0.993. The adjusted . R‐Square value of the fitted graph was 0.985. f) Piezoelectric output current with applied pressure over the range of 40 Pa to 113 kPa (*n =* 5). g) Output current response from the interlocked e‐skin under simultaneous pressure (40 Pa–113 kPa) and ∆*T* (0.04−1.8 K) (*n =* 5). h) Piezoelectric response time as a function of temperature. The inset shows the normalized piezoelectric output current peaks measured under different ∆*T*. i) Pressure‐dependent piezoelectric relaxation time measured under a range of ∆*T* of 0−1.8 K.

The higher level of polarization confinement in the interlocked microdome than in the other microstructures indicates that the serpentine interlocked region in the microdome structures is more favorable for enhancing the polarization than the other interlocked microstructures (micropillar and micropyramid), where weaker van der Waals forces due to higher d‐spacing in the intermolecular packing limit electron cloud polarization. As a result, the interlocked microdome nanogenerator showed superior piezoelectricity in comparison to other structured nanogenerators. The effective piezoelectric coefficient that contributes to the energy harvesting process can be evaluated using the quasi‐static method as, $|d_{\mathrm{eff}}|=\frac{Q}{F}$, where the generated charge (*Q*) was estimated by integrating the output current profile under the pushing force (*F*) of 11.3 N (i.e., 113 kPa) (Figure S17, Supporting Information).^[^ ^32^ ^]^ The interlocked microdome e‐skin nanogenerator had as *d*_eff_ of 19.6 ± 0.2 pC N^−1^, which indicates ≈82% utilization of converse *d*_33_ of ≈24 pC N^−1^ in the energy harvesting process. The *d*_33_ value of the interlocked gelatine microdome was higher than that of several biodegradable,^[^ ^33^, ^34^, ^35^, ^36^, ^37^ ^]^ and even non‐biodegradable organic^[^ ^38^ ^]^ and inorganic materials,^[^ ^39^, ^40^, ^41^ ^]^ reported to date (**Figure** **3**a). A detailed comparison of the materials is given in Table S4, Supporting Information. Additionally, the interlocked microdome nanogenerator had a much higher level of pyroelectricity than the planar (Figure 3b) and other microstructured ones (Figures S18, S19a, Supporting Information). The interlocked microdome nanogenerator generated an output current of ≈0.03 nA cm^−2^ in response to a very small Δ*T* of 0.04 K (Figure 3c), which is 10 times smaller than the minimum temperature detection limit (≈0.4 K) of PZT (lead zirconate titanate) micro/nanowires.^[^ ^42^ ^]^ This minimum detection limit of Δ*T* = 0.04 K was not achievable with planar or other microstructured nanogenerators, which had a minimum Δ*T* of 0.3 K (Figure S18, Supporting Information). The interlocked microdome nanogenerator had a temperature sensitivity of ≈2.6 µA m^−2^K^−1^, which is 13 times higher than that of the planar one (≈0.2 µA m^−2^K^−1^) (Figure S19b, Supporting Information). Importantly, the stable pyroelectric current generation over 1300 repeated cycles demonstrated the reliable and stable thermal sensing performance of the nanogenerator, even under higher Δ*T* ≈ 1.8 K and $\frac{dT}{dt}$ ≈ 0.4 K s^−1^ (Figure S20, Supporting Information). The linear curve in Figure S21, Supporting Information was used to determine a pyroelectric coefficient (*Π*) of 13 µC m^−2^K^−1^ for the interlocked microdome nanogenerator based on the relationship $I_{\mathrm{pyro}}=\;\Pi A\frac{dT}{dt}$,^[^ ^28^ ^]^ where *A* is the electrode area; this value was 11.8 times higher than that (≈1.1 µC m^−2^K^−1^) of the planar nanogenerator. The excellent *Π* of our interlocked e‐skin nanogenerator compares favorably with previous biodegradable and even non‐biodegradable organic and inorganic pyroelectric nanogenerators (Figure 3d, Table S5, Supporting Information).^[^ ^43^, ^44^, ^45^, ^46^, ^47^, ^48^, ^49^, ^50^, ^51^, ^52^, ^53^ ^]^

In addition to pyroelectric temperature sensing, the interlocked gelatine microdome nanogenerator showed piezoelectric pressure sensitivity ($S\;=\frac{\Delta V_{\mathrm{oc}}}{\Delta \sigma }$) of 1 and 0.026 mV Pa^−1^ for the pressure ranges of 40−100 Pa and 100 Pa−100 kPa, respectively, which are higher than those of other microstructures (Figure S22, Table S6, Supporting Information). In particular, in the ultralow pressure range of 0.005−1.7 Pa (Figure 3e), the interlocked microdome nanogenerator showed excellent sensitivity of 41 mV Pa^−1^. The pressure sensitivity below 2 Pa was explored using acoustic waves (Figure S23, Note S3, Supporting Information). Furthermore, the gelatine microdome e‐skin nanogenerator was capable of detecting pressures as low as 0.005 Pa (detection limit), which were not detectable by other nanogenerators (Figure S24, Table S6, Supporting Information) and even exceeds the typical sensitivity of human skin.^[^ ^54^ ^]^ Our biodegradable gelatine e‐skin nanogenerator was comparable to the state‐of‐the‐art PZT sensors in terms of detection limit (≈0.005 Pa, the lowest possible detection limit by piezoelectric sensor)^[^ ^55^ ^]^ and pressure sensitivity (≈0.82^[^ ^55^ ^]^ and 80 µV Pa^−1[^ ^56^ ^]^). A detailed comparison in Table S7, Supporting Information indicates the outstanding pressure sensing performance of our biodegradable gelatine e‐skin nanogenerator over previous non‐biodegradable nanogenerators. Therefore, the gelatine e‐skin nanogenerator could detect very light natural objects, such as a flower (≈85 mg), leaf (≈7 mg), and even a hair (≈0.5 mg) (Figure S25, Supporting Information). In addition, the piezoelectric output current of the interlocked microdome nanogenerator was superior to the planar (Figure 3f) and other microstructured ones (Figure S26, Supporting Information). The non‐linear variation in output response is attributed to the pressure‐induced hardening of gelatine with the reversible coil‐helix transition, where mechanical strain was non‐linearly generated under compressive stress (Figure S27, Note S4, Supporting Information).^[^ ^57^ ^]^ Importantly, the stable piezoelectric output response over 2 months without any significant degradation (Figure S28, Supporting Information) proves the reliability of the interlocked nanogenerator for e‐skin applications.

The pyroelectric‐coupled piezoelectric output current under a range of ∆*T* (≈0−1.8 K) and *σ* (≈40 Pa−113 kPa) conditions are shown in Figure 3g and Figure S29, Supporting Information. Benefitting from the pyroelectric coupling, the piezoelectric output current increased with increasing ∆*T*, resulting in enhanced pressure sensitivity (Figure S30, Supporting Information). In addition, our gelatine nanogenerator showed ultrafast detection of pressure, with a response time as low as 1.9 ms (Figure S31a,b, Supporting Information) within the ultralow pressure region (<2 Pa) and 32 ms within the subtle to medium pressure range (42 Pa–100 kPa, Figure S31c, Supporting Information). In contrast to the piezoelectric response time, the interlocked nanogenerator had a slightly longer pyroelectric response time of 333 ms (Figure S31d, Supporting Information) in the range of ∆*T* ≈ 0.04–1.8 K, which is still faster than previous pyroelectric PZT micro/nanowires (≈0.9 s)^[^ ^42^ ^]^ and BaTiO_3_ (≈8.6 s) devices.^[^ ^58^ ^]^ During the simultaneous detection of temperature and pressure, while the piezoelectric response time increased non‐linearly (Figure 3h) with increasing ∆*T* ≈ 0.04–1.8 K due to the pyroelectric coupling, the response time remained constant for a particular ∆*T* over the pressure range of 40 Pa to 100 kPa (Figure S32, Supporting Information). The non‐linearity of the response time is described by *τ*_R_ =  *a*Δ*T^b^* (ms), where *a =* 177 and *b =* 0.3 are the power law fitting parameters (from Figure 3h). Importantly, this graph and equation can be used to calibrate the device to quantify the temperature of an unknown object. Additionally, the relaxation/decay time of the pressure response (Figure S33a, Supporting Information) decreases asymptotically with increasing pressure (Figure 3i). It implies that piezoelectric charges decay slowly at higher temperature, which is consistent with a previous observation.^[^ ^59^ ^]^ The pyroelectricity‐induced dipole randomization delays the polarization ordering under pressure, which increases the response time.^[^ ^28^, ^60^ ^]^ For a particular ∆*T* with a constant dipole randomization, the increase in the polarization gradient in response to the applied pressure causes a rapid charge decay and decreases the relaxation time. The piezoelectric charge decay process is faster than that of the pyroelectric process (Figure S33b, Supporting Information). The pressure and temperature can be correlated with the relaxation time by fitting the graphs with exponential functions (Figure S34, Supporting Information),

(1) $$\tau_{r}=f\left(P,\Delta T\right)=\tau_{0}\;\left(\Delta T\right)+\sum_{i=1}^{3}A_{i}\left(\Delta T\right)e^{\left(-\frac{P}{T_{i}\left(\Delta T\right)}\right)}$$

where *τ*_0_(Δ*T*), *A_i_*(Δ*T*), and *T_i_*(Δ*T*) are the Δ*T* − dependent constants (see Note S5, Supporting Information). This empirical formula is similar to Saint–Venant's principle.^[^ ^61^ ^]^ Using this formula, we can easily identify the pressure and temperature of an unknown object in contact with the e‐skin nanogenerator.

### 2.3 Wearable Device Applications of the Gelatine Skin Sensor

**Figure 4:**
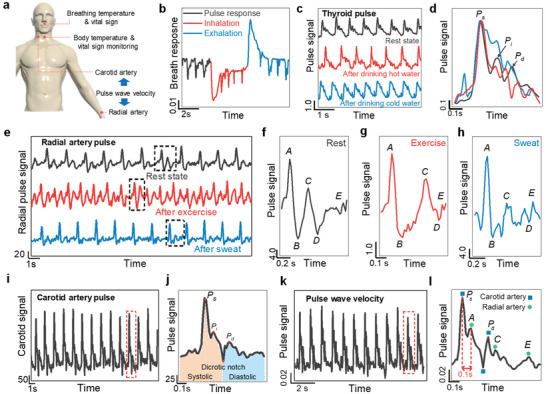
Physiological signal monitoring of the gelatine skin sensor. a) Schematic describing the health status monitoring possibilities using our developed e‐skin. b) Output response from the e‐skin during breathing (inhalation and exhalation). c) Arterial pulse measurement under rest conditions, and after drinking hot or cold water, with d) an enlarged view for each pulse cycle. e) Wrist pulse signals under different physiological conditions with an enlarged view of a corresponding single cycle: f) rest, g) exercise, and h) sweating conditions. Each cycle consists of four waves with a systolic nature: *A*‐wave (initially positive), *B*‐wave (early negative), *C*‐wave (re‐increasing), *D*‐wave (late re‐decreasing); and one wave of diastolic nature: *E*‐wave (positive). i) Carotid artery pulse signal measurement and j) an enlarged view of one cycle showing typical morphological characteristics. k) Simultaneous measurement of carotid artery and radial artery pulse signals for PWV measurement (as shown by the schematic in the inset) with l) an enlarged view of one cycle.

With the above decoupling capability of piezoelectric and pyroelectric signals, the gelatine e‐skin nanogenerator is able to simultaneously monitor vital signs, including the body temperature and pulse pressure (**Figure** **4**a). When the e‐skin nanogenerator was attached to the philtrum of the human face, it was able to monitor the heart rate (62 beats per minute) and identify inhalation (negative peak) and exhalation (positive peak) processes (Figure 4b). In particular, using the coupled pyro/piezoelectric relationship (Equation (1)), the Δ*T* during breathing was simultaneously calculated to be 6 K (Figure S35, Supporting Information), which is consistent with the measured value (Figure S36, Supporting Information) and reported literature values.^[^ ^47^, ^56^ ^]^ Additionally, the e‐skin nanogenerator was capable of discriminating different physical activities affecting the cardiovascular system. For example, an e‐skin nanogenerator attached to the throat (Figure S37a, Supporting Information) displayed the thyroid pulse waveform, where significant changes in heart rate, augmentation index, and reflection index values were observed after drinking hot and cold water (Figure 4c) due to the vasoconstriction and vasodilation of blood vessels induced by cold and heat stresses, respectively (Figure 4d; Figure S37b–d, Supporting Information).^[^ ^62^ ^]^ In addition to the pulse detection, swallowing of saliva, coughing, and drinking actions by a healthy young man were precisely identified via glottis opening and closing processes during the movement of the laryngeal prominence (the Adam's apple) (Figure S37e–i, Supporting Information). Importantly, the biocompatibility of the gelatine e‐skin nanogenerator causes no damage, allergic reactions, or redness to the skin after continuous attachment for 6 h (Figure S38a,b, Supporting Information). This allows the continuous attachment of the e‐skin nanogenerator on the wrist for the monitoring of the arterial pulse waveform, which enables the discrimination of resting, exercising (jogging for 5 min), and sweating conditions (Figure 4e–h) (Note S6, Supporting Information). The e‐skin nanogenerator was able to accurately reproduce the pulse waveform (Figure 4e,h) even under exposure to artificial sweat, demonstrating its versatility for use with sweaty patients (Figure S38c, Supporting Information). In our nanogenerator, the cross‐linked gelatine thin films are used as top and bottom substrates (Figure S1c, Supporting Information), which effectively protect the Mg electrode layers against corrosion by sweat or other humid environments.

The e‐skin nanogenerator was also able to detect the carotid artery pulse wave (Figure 4i) with typical morphological features (Figure 4j, Note S7, Supporting Information) when attached to the neck of a volunteer (Figure S39, Supporting Information). The cardiovascular parameters (Notes S6,S7, Supporting Information) were consistent with the literature values.^[^ ^63^ ^]^ The pulse wave velocity (PWV) was measured by attaching multiple e‐skin nanogenerators simultaneously to different artery sites, including the wrist and carotid arteries (Figure 4k). The enlarged view of a single pulse cycle evidences the superposition of radial pulse waves and carotid artery pulse waves (Figure 4l). The carotid artery pulse wave (main wave, *P*_s_) was detected 100 ms earlier than the radial artery pulse wave (*A*‐wave), resulting in a PWV of 5.8 ms^−1^, which is consistent with the typical aortic PWV obtained by conventional clinical instruments and state‐of‐the‐art self‐powered sensors.^[^ ^63^ ^]^

**Figure 5:**
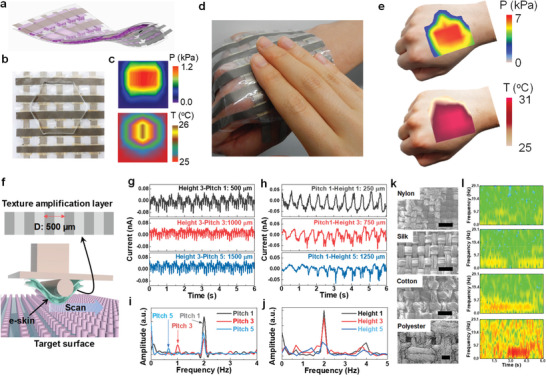
Prosthetic arm and texture perception applications of the gelatine skin sensor. a) Schematic of the flexible 5 × 5 pixel sensor array matrix. b) Photograph of the sensor network with an irregular object placed on it to map the c) spatial distribution of pressure (*P*) and temperature (*T*) upon contact. d) The wearable sensor network patch (representing a prosthetic arm) touched by another human hand could spatially resolve the e) pressure and temperature distribution. f) Schematic of the texture perception setup using time‐dependent output currents from e‐skin obtained by scanning the micropillar surfaces with different g) pitch size and h) height with their respective FFT patterns shown in i,j), respectively, to discriminate the surface texture variations. k) SEM images of commercial textile fabrics (scale bar: 500 µm) used for surface smoothness recognition through l) STFT patterns of the output currents generated by scanning the e‐skin over them.

For applications in wearable tactile devices requiring simultaneous pressure and temperature sensing capability, a gelatine sensor array (5 × 5 pixel) was fabricated (**Figure** **5**a). When in contact with an object (Figure 5b), the sensor array demonstrated an excellent mapping of the contact pressure (0–1.2 kPa) and temperature (25–26 °C, ∆*T* = 1 °C) (Figure 5c). Owing to its conformability and flexibility, the sensor array can be worn on the hand (Figure 5d) and can collect and map the pressure (0–7 kPa) and temperature (25–31 °C) distribution when another hand touches the sensor array (Figure 5e). These demonstrations demonstrated the excellent spatial resolution of simultaneous temperature and pressure measurements using our bi‐functional gelatine sensor array, which could be used in robotic skin and prosthetic limb applications. The human fingertip skin can distinguish the surface texture by touching and lateral sliding. Similarly, our gelatine e‐skin nanogenerator was used in haptic perception for surface texture recognition by lateral sliding (scanning speed ≈ 1 mm s^−1^; force ≈ 0.098 N) (Figure 5f). A fingerprint‐like pattern with a grating period of 500 µm was used as an upper layer on the gelatine e‐skin nanogenerator as a texture amplifier.^[^ ^64^ ^]^ The fast Fourier transformation (FFT) of the output currents (Figure 5g,h) from the e‐skin nanogenerator can easily discriminate the texture variation of 3D‐printed micropillar arrays with different pitch sizes and heights (Figure 5i,j; Figure S40, Supporting Information). With increasing pitch size, the FFT peak position shifted to the low frequency region with the presence of a fundamental frequency at the same position (Figure 5i). The shifted peaks matched well with the pitch sizes, which can be calculated by the scanning speed and frequency. The fundamental frequency ($f\;=\frac{\nu }{\lambda }\;=\;2\;\mathrm{Hz}$) was calculated from the texture amplifier grating period (*λ*) and the scanning speed (*ν*). Furthermore, the peak intensity of *f* in the FFT reduces with increasing pillar height (Figure 5j) due to the corresponding reduction in the effective modulus of the surface pillars.^[^ ^64^ ^]^ In contrast, the planar gelatine e‐skin nanogenerator with the texture amplifier layer was not able to discriminate the surface texture variation (Figure S41, Supporting Information). The gelatine e‐skin nanogenerator was further used for the perception of surface smoothness of commercial knitted textile fabrics, such as polyester, cotton, silk, and nylon (Figure 5k). The short‐time Fourier transform (STFT) patterns of the output currents (Figure S42, Supporting Information) were used to distinguish time‐dependent roughness features for different fabrics (Figure 5l). For example, the rougher surface produced high‐intensity signals of all frequencies below 30 Hz. Accordingly, the smoothness of the fabrics was detected in the order of nylon > silk > cotton > polyester.

## 3 Conclusion

With the aim of producing sustainable and eco‐friendly electronic devices without any e‐waste footprint, we demonstrated a fully biodegradable and ferroelectric gelatine e‐skin nanogenerator with the ability to simultaneously sense temperature, pressure, and surface texture variations. We showed that the physically confined gelatine within the interlocked microdome structure significantly enhanced the polarization and ferroelectric properties. Our gelatine e‐skin nanogenerator displayed excellent sensing capabilities to simultaneously detect and discriminate the temperature and pressure variations with the lowest detection limit ever reported. Proof‐of‐concept demonstrations of our gelatine e‐skin nanogenerator toward healthcare monitoring, simultaneous temperature–pressure mapping, and texture perception verified that it has great potential for applications in environmentally benign wearable sensors, robotic skins, and prosthetic limbs. Furthermore, our gelatine e‐skin nanogenerator, composed of active gelatine extracted from porcine skin and Mg electrodes, was biodegradable and fully decomposed in a body fluid environment within a month, demonstrating an ideal recycling process of decomposable transient electronics with zero waste footprint. Our devices have numerous advantages, such as excellent ferroelectric properties, low‐cost fabrication, edibility, and biodegradability, and are based on a simple yet powerful design strategy. Hence, we expect that these devices could be useful components in current electronic devices (such as sensors, actuators, transistors, memory, and capacitors) and the future internet‐of‐everything devices including wearable, implantable, and decomposable devices that do not generate any pollution or e‐waste.

## 4 Experimental Section

##### Film Preparation

Gelatine powder (Type A, porcine skin, gel strength ≈ 300 g Bloom; Sigma Aldrich) was mixed with deionized water to prepare 30 w/v gelatine solution, which was stirred at 60 °C for 24 h. To fabricate the micropatterned gelatine films, inverse polydimethylsiloxane (PDMS) moulds were prefabricated from Si moulds which were produced by conventional photolithographic techniques with a dry etching process and coated with a self‐assembled monolayer of FOTS (1H,1H,2H,2H‐perfluorooctyltrichlorosilane) (AVC‐150M, SORONA, Korea) as an anti‐adhesion layer for the easy demoulding of hierarchical micropatterned PDMS. Then, the gelatine solution was spin coated (≈1000 rpm for 90 s) on an oxygen plasma treated (≈3 s) inverse micropatterned PDMS substrate. Then, the gelatine was dried at 40 °C under a range of humidity conditions (40–80%) for 24 h to prepare several different gelatine films. Then, the gelatine films were peeled off the substrate and cross‐linked with GA solution (25% in H_2_O, Sigma Aldrich). The cross‐linking was performed in a vacuum chamber with a saturated vapor pressure of GA solution for 24 h. Finally, the cross‐linked gelatine film was used for characterization and device fabrication. As a reference, a planar gelatine film without any micro‐patterning was also prepared using the same procedure.

##### Device Fabrication

The interlocked micropatterned devices were fabricated by placing two films with their patterned sides facing each other. The Mg electrode was deposited on the non‐patterned sides of the gelatine films using DC sputtering (SRN‐120, SORONA). Then, Cu–Ni plated adhesive conducting fabric was attached to the edge side of electrodes with silver paste and dried at room temperature. The fabricated device was electrically poled using an electric field of 100 MVm^−1^ for 3 h with a high‐voltage power supply (PS350, Stanford Research System). Then, the interlocked films were encapsulated by planar gelatine films using a spray bandage.

##### In Vitro Biodegradation

After immersion of e‐skin in PBS solution, the e‐skin was taken out of solution after specific intervals and rinsed with distilled water to avoid any error at weight measurement caused by remained salt particles of PBS. The e‐skin was placed in the filter paper to soak any remaining water and weighted to evaluate their weight loss due to degradation. The percentage of weight loss was calculated using the following relation, Degradation (%) = $\frac{w_{i}-w_{f}}{w_{i}}\times \;100$, where *w*_i_ and *w*_f_ are the initial and final weights of the e‐skin after biodegradation at specified time interval.

##### Characterization

The surface morphology was observed by scanning electron microscopy (SEM; S‐3400N, Hitach High‐Technologies). The structural properties were studied by XRD (D/MAX2500V/PC, RIGAKU) and FT‐IR (670‐IR, Varian). Ferroelectric hysteresis loops were measured using a Radiant Technology ferroelectric tester (Precision LC II) connected to a probe station (MS TECH, MODEL 8000) with an optical set up (PSM 1000) (Figure S43, Supporting Information). The output voltage was measured using a digital phosphor oscilloscope (DPO 2022B), while the current was measured using a source meter (S‐2400, Keithley). A pressure was applied to the e‐skins with a pushing tester (JIPT, Junil Tech). The acoustic response was measured using a loudspeaker test oscillator (SG‐3428B, Sigma Eltec) and the sound pressure level was measured using a sound level meter (TENMARS, TM‐102). The external temperature oscillations were applied to the sensors by an IR bulb modulated by a frequency generator (AFG3011C, Tektronics). The time‐dependent temperature was recorded in real‐time as well as simultaneously with current measurement via an online interface with computer programmed LabVIEW software using a K‐type thermocouple connected to a temperature data logger (Pico Technology USB TC‐08). Thermal images were captured by an Android‐based infrared camera (Therm App). FEM simulations were performed using COMSOL Multiphysics software. Artificial sweat (pH 4.5) was used for healthcare monitoring (Artificial Eccrine Perspiration 1700‐0020, Pickering pH 4.5). 3D‐printed micropillar arrays used for texture perception were prepared using a stereo lithography apparatus 3D printer (SINDOH A1).

## Conflict of Interest

The authors declare no conflict of interest.

## Author Contributions

S.K.G and H.K. conceived the research. S.K.G. and J.P. designed the experiments. S.K.G. and J.P. performed the experiments. S.K.G. and S.N. performed material characterizations. M.P.K. carried out the ferroelectric measurement. S.K.G. and H.K. wrote the manuscript. All of the authors provided feedback and approved for final submission.

## Supporting information

Supporting Information

## Data Availability

Research data are not shared.
