# Supplementary material for: A Fully Biodegradable Ferroelectric Skin Sensor from Edible Porcine Skin Gelatine
Source: Adv Sci (Weinh). 2021 May 7;8(13):2005010. doi: 10.1002/advs.202005010 (PMC8261503; doi:10.1002/advs.202005010)
Supplement: Supplementary file 1 — Supporting Information [file ADVS-8-2005010-s001.pdf]

© 2021 Wiley-VCH GmbH

ADVANCED SCIENCE
Open Access

Supporting Information

for *Adv. Sci.,* DOI: 10.1002/advs.202005010

A Fully Biodegradable Ferroelectric Skin Sensor from Edible Porcine Skin Gelatine

*Sujoy Kumar Ghosh, Jonghwa Park, Sangyun Na, Minsoo P. Kim and Hyunhyub Ko**

Supporting Information

A Fully Biodegradable Ferroelectric Skin Sensor from Edible Porcine Skin Gelatine

Sujoy Kumar Ghosh, Jonghwa Park, Sangyun Na, Minsoo P. Kim and Hyunhyub Ko[*]

School of Energy and Chemical Engineering, Department of Energy Engineering, Ulsan National Institute of Science and Technology (UNIST), Ulsan Metropolitan City 44919, Republic of Korea

*E-mail: hyunhko@unist.ac.kr

Note S1. FT-IR

As shown in Supplementary Fig. 8a, considering the –CONH group of the peptide chain, all of the films showed FTIR peaks attributed to the stretching vibration of –C=O (amide I band), bending of N–H (δ(N–H)) coupled with stretching of –C–N (ν(C-N)) (amide II band), and bending of N–H (δ(N–H)) (amide III band). The amide I band was observed around 1643 cm^{-1} for the planar gelatine film, whereas this band was continuously shifted towards higher wavenumbers for microstructured gelatine films (~1645 cm^{-1} for the micropillar and micropyramid films, and ~1648 cm^{-1} for the microdome film). Furthermore, the shifting of the amide III band towards higher wavenumbers was only significant for the microdome film. For instance, the amide III band was observed around 1238 cm^{-1} for planar and micropyramid structured gelatine films, whereas, it was shifted towards 1240 cm^{-1} for micropillar and microdome patterns. The maximum shift in the amide I and III bands was observed for the microdome film, which indicated the presence of more stretched –C=O and N–H bonds of –CONH hydrogen bonding motifs in the microdome structure.

In addition, the absorption intensity ratio of the amide II band (1539 cm^{-1} for planar, micropillar, and microdome; 1529 cm^{-1} for micropyramid) to the $\delta(CH_2)$ band (1450 cm^{-1} for planar; 1446 cm^{-1} for micropillar and microdome; 1444 cm^{-1} for micropyramid) (Supplementary Fig. 8b) was around 1.0, indicating the confined triple helical structure of collagen. However, this ratio was slightly higher for the microstructured gelatine films (1.26, 1.29, and 1.32 for the micropillar, micropyramid and microdome structures) than for the planar (~ 1.19), as shown in Supplementary Fig. 8b.

Note S2. Device simulations

Vorticity and viscous stress simulation

The vorticity and viscous stress distribution of the spinning solution within several microstructured and planar substrates was computed using "Laminar Flow interface". The Coriolis (~ $2m\omega u$) and centrifugal (~ $m\omega^2 r$) forces acted on the fluid of mass m during rotational motion of the fluid substrate with angular velocity ω and radius r. Here, $\boldsymbol{u}$ is the velocity of the fluid. During spin coating, the centrifugal force induces a parabolic flow profile in the radial direction. This is analogous to a Poiseuille or pressure-driven flow. The velocity-dependent Coriolis force produces an inhomogeneous transverse force in the tangential direction, which has its highest value in the centre of the fluidic substrate. This results in a change in the vorticity and viscous stress distribution from that of a standard Poiseuille flow. Here, the Navier Stokes momentum equation was used to solve the fluid velocity dynamics.

$$\rho(\boldsymbol{u}.\nabla)\boldsymbol{u} = \nabla.[-p\mathbf{I} + \boldsymbol{K}] + \mathbf{F} \qquad \text{(S1)}$$

$$\nabla.(\rho\boldsymbol{u}) = 0 \qquad \text{(S2)}$$

$$\boldsymbol{K} = \mu(\nabla\boldsymbol{u} + (\nabla\boldsymbol{u})^T) - \frac{2}{3}\mu(\nabla.\boldsymbol{u})\mathbf{I} \qquad \text{(S3)}$$

Here, $p\mathbf{I}$ is the volumetric stress where $\mathbf{I}$ is the identity tensor, ρ is the density, μ is the dynamic viscosity and $\boldsymbol{u}$ is the velocity of the fluid. In this equation, $\nabla\boldsymbol{u} + (\nabla\boldsymbol{u})^T$ represents the rate-of-strain tensor, and $\nabla.\boldsymbol{u}$ is the rate of expansion of the flow. Thus, viscous stress was calculated from equation S3. Then, by solving equations S2 and S3, $\boldsymbol{u}$ was calculated to obtain the vorticity. The vorticity was calculated by taking the curl of the Navier Stokes momentum equation, which gives equation S4.

$$\frac{D\boldsymbol{\omega}}{Dt} = (\boldsymbol{\omega}.\nabla)\boldsymbol{u} + \mu\nabla^2\boldsymbol{\omega} \qquad \text{(S4)}$$

Piezo/pyroelectric simulations

The finite element method (FEM)-based simulation was performed using a temperature variation ΔT ~ 1.8 K and pressure of 113 kPa using COMSOL Multiphysics software.

The piezoelectric simulation was performed using "Piezoelectric Effect (pze)" module. We solved the linear mechanical equation S5 that relates the stress $\boldsymbol{T}$ to the applied force F on the device and the Poisson equation S6 that relates the electric displacement $\boldsymbol{D}$ to the fixed charge density ρ_V:

$$-\nabla.\boldsymbol{T} = F \;, \qquad \text{(S5)}$$

$$\nabla.\boldsymbol{D} = \rho_V \;, \qquad \text{(S6)}$$

The coupling between the structural and electrical domains can be expressed in the form of a connection between the material stress and its permittivity at constant stress or as a coupling between the material strain and its permittivity at constant strain. The equations S5 and S6 are coupled to the piezoelectric equations of strain-charge form (equations S7, S8) and stress-charge form (equations S9, S10) that correlate the stress tensor $\boldsymbol{\sigma}$, strain $\boldsymbol{S}$, electric displacement $\boldsymbol{D}$, and the electric field $\boldsymbol{E}$ using the permittivity $\boldsymbol{\varepsilon}$, elasticity tensor $\boldsymbol{c}$, and piezoelectric coupling tensors $\boldsymbol{e}$ and $\boldsymbol{d}$.

Strain-Charge form:

The strain-charge form of a piezoelectric material is written as:

$$\boldsymbol{S} = s_E.\boldsymbol{\sigma} + d^T.\boldsymbol{E} \qquad \text{(S7)}$$

$$\boldsymbol{D} = \boldsymbol{d}.\boldsymbol{\sigma} + \varepsilon_0.\varepsilon_{rT}\boldsymbol{E} \qquad \text{(S8)}$$

The material parameters s_E, d, and ε_{rT} correspond to the material compliance, coupling properties, and relative permittivity at constant stress, respectively, and ε_0 is the permittivity of free space.

Stress-Charge form:

The stress-charge form of the piezoelectric material is written as:

$$\boldsymbol{\sigma} = c_E.\boldsymbol{S} + e^{\sigma}.\boldsymbol{E} \qquad \text{(S9)}$$

$$\boldsymbol{D} = e.\boldsymbol{S} + \varepsilon_0.\varepsilon_{rS}.\boldsymbol{E} \qquad \text{(S10)}$$

The material parameters c_E, e, and ε_{rS} correspond to the material stiffness, coupling properties, and relative permittivity at constant strain, respectively.

A pyroelectric simulation was performed using Multiphysics coupling of two modules, namely, “Temperature Coupling (tc)” and “Piezoelectric Effect (pze)”. The Multiphysics interface uses the continuity equation (S11), momentum equation (S12), and the heat transfer in a solid equation (S13):

$$\frac{\partial \rho}{\partial t} + \nabla \rho \boldsymbol{u} = 0 \qquad \text{(S11)}$$

$$\rho \frac{\partial u}{\partial t} + \rho \boldsymbol{u}.\nabla \boldsymbol{u} = -\nabla \sigma + \boldsymbol{F} \qquad \text{(S12)}$$

$$\rho C_P \left(\frac{\partial T}{\partial t} + (\boldsymbol{u}.\nabla)T\right) = -(\nabla.q) - \boldsymbol{Q} \qquad \text{(S13)}$$

Here, ρ is the density, $\boldsymbol{u}$ is the velocity, $\boldsymbol{F}$ is the device force vector**,** C_P is the specific heat capacity, q is the heat flux by conduction, and $\boldsymbol{Q}$ is the viscous heating of a periodic heat source. The simulations of the gelatine films were conducted using the materials parameters shown in Supplementary Table 3.

Note S3. Acoustic sound pressure

The pressure sensitivity below 2 Pa was investigated using acoustic sound waves (Supplementary Fig. 23a) with the well-established acoustic sensing units shown in Supplementary Fig. 23b. The pressure applied by the sound waves was calculated using $\sigma_a = \sigma_0 10^{\frac{L_p}{20}}$, where, σ_0 is the standard reference sound pressure of 20 μPa, and L_p is the measured sound pressure level. Here, the pressure sensitivity was evaluated at the sound frequency of 200 Hz because the devices showed the maximum output voltage at this resonance frequency (Supplementary Fig. 23c). Additionally, the highly responsive interlocked microdome pattern device was able to detect the high-frequency vibration of acoustic sounds of the recorded phrase “*Biodegradable electronic skin*” generated by a commercial speaker (Supplementary Fig. 23d). The short-time Fourier transform (STFT) spectrogram of the sound source ranging from 0−4 kHz matched well with the STFT of output voltage signals from the interlocked microdome device. In contrast, the single planar device could not precisely monitor the acoustic signals.

Note S4. Relationship between the mechanical and electrical properties of the interlocked microdome device

The non-linearity in the electrical output response arises due to the compressive strain hardening property of gelatine, resulting in the non-linear mechanical properties of the

interlocked gelatine device, as shown in Supplementary Fig. 27a, where the generated strain (ε) non-linearly increases with increasing compressive stress (σ). In contrast, the piezoelectric V_{oc} and I_{sc} linearly depend on the generated strain under applied compressive stress, following $V_{oc} = \frac{d_{33} Y_c A \varepsilon}{C_p}$; $I_{sc} = \frac{dq}{dt} = d_{33} Y_c A \dot{\varepsilon}$, where C_p is the capacitance and Y_c is the compressive modulus of the e-skin. As a result, the output voltage and current exhibit a non-linear increment with increasing pressure. The non-linearity of the mechanical stress–strain relationship below 10 kPa can be expressed as $\sigma = Y_c \varepsilon$ (0.2 < ε < 0.5) and $\sigma = K\varepsilon^{\alpha}$ (0.5 < ε < 0.8), where K is related to the compressive modulus and α is the strain hardening exponent and representative to the non-linear behaviour, which was obtained from fitting the experimental data with a power law relationship (Supplementary Fig. 27b,c). In this case, the obtained α ~ 5.4 is similar to a previously reported value.[1] This strain hardening behaviour is attributed to the stress-induced coil-helix transition of gelatine. As the renaturation of protein molecules is completely reversible, gelatine is able to recover its original structure after stress removal, displaying its intrinsic non-linear elastic nature.

Note S5. Correlation between pressure and temperature

The empirical relationship between pressure and temperature is written as:

$$\tau_r = f(P, \Delta T) = \tau_0(\Delta T) + \sum_{i=1}^{3} A_i\,(\Delta T) e^{\left(-\frac{P}{T_i(\Delta T)}\right)}$$

$$\tau_r = f(P, \Delta T) = \tau_0 + Ae^{-\frac{P}{T_1}} + Be^{-\frac{P}{T_2}} + Ce^{-\frac{P}{T_3}}$$

Here, $A_1 = A;\ A_2 = B;\ A_3 = C$ are considered for convenience. The unknown parameters were obtained from $\tau_0 = \tau_1 - \tau_2 e^{\Delta T \alpha}$ (obtained by fitting the data points shown in Fig. 3i), where, constants $\tau_1 = 615$, $\tau_2 = 360$, and $\alpha = 2.3$ was obtained by fitting the τ_0 values for each ΔT~ $0.04\ K - 1.8\ K$. Similarly, the other parameters were obtained as follows:

$A = a_1 - a_2 e^{-\Delta T \beta}$, where, the constants $a_1 = 149$, $a_2 = 97$, and $\beta = 1.5$

$B = -b_1 + b_2 e^{\Delta T \gamma}$, where, the constants $b_1 = 283$, $b_2 = 143$, and $\gamma = 2.8$

$C = c_1 - c_2 e^{-\Delta T \delta}$, where, the constants, $c_1 = 105$, $c_2 = 97$, and $\delta = 7.1$

The additional parameters are described as follows:

$$T_1 = t_{10} - t_{11} e^{-\Delta T \varphi_1}$$

$$T_2 = t_{20} \Delta T - t_{21}$$

$$T_3 = t_{30} - t_{31} e^{-\Delta T \varphi_2}$$

Here, $\tau_1 = 615$, $\tau_2 = 360$, $\alpha = 2.3$, $t_{10} = 1204$, $t_{11} = 1104$, $\varphi_1 = 5$, $t_{20} = 64776$, t_{21}=13800, $t_{30} = 46$, $t_{31} = 48$, and $\varphi_2 = 2.2$.

Note S6. Arterial pulse monitoring

The radial artery pulse waves were detected by the *e*-skin while the volunteer was in a resting state, after exercise (jogging for 5 min), and while sweating (Fig. 4e in the main manuscript). The measured pulse rate was 69 BPM at rest and 105 BPM after physical exercise. The morphological characteristic of the arterial pulse waveform is entirely different from that of the thyroid and philtrum area arterial pulse. For all physical conditions (Fig. 4f-h), the pulse waveform is composed of four waves of systolic nature, i.e. *A*-wave (initially positive), *B*-wave (early negative), *C*-wave (re-increasing), *D*-wave (late re-decreasing), and one wave of diastolic nature, i.e. *E*-wave (positive). In this case, the ratio of peak heights such as *E*-, *D*- and *C*- waves to *A*- wave (i.e., $P_{A/E}$, *C*/*A*, and *D*/*A*, respectively) and the time delay (τ_{EA}) between the *A*- and *E*-wave were very prominent for the rest state ($P_{A/E}$~ 8.0; $C/$

A~ 0.21; D/A~ − 1.0; τ_{EA}~ 0.8) (Fig. 4f) and after exercise ($P_{A/E}$~ 9.0; C/A~ 0.6; D/A~ − 1.4; τ_{EA}~ 0.48) (Fig. 4g), and are the key indicators of any diastolic dysfunction, leading to the symptoms of heart failure. Furthermore, the performance of the *e*-skin for the radial artery pulse wave measurement was similar (BPM: 69, $P_{A/E}$ ~ 5.0, *C/A* ~ 0.2, *D/A*~ −0.5 and τ_{EA}~ 0.6,) after 1 h exposure to sweat (Fig. 4h).

Note S7. Discussion of carotid artery pulse signals

The single pulse cycle of the carotid artery signal is depicted in Fig. 4j (main manuscript). It consists of the systolic phase and diastolic phase, the junction of which is called the dicrotic notch. In the systolic phase, the blood pumping from the heart results in a sharp rise in blood pressure, corresponding to the main wave (P_s) in the pulse waveform. The predicrotic wave (P_i) is formed by the multiple reflections from the arterial wall. In the diastolic phase, the elasticity of the vessels causes the small amount of blood pumping back to the heart to produce a brief rise in blood pressure, appearing as the dicrotic wave (P_d). During carotid artery pulse wave measurement, the measured pulse rate was 84 BPM, which was similar to the value measured for the thyroid arterial pulses. The value of AI_a~ -0.78 was close to the systolic augmentation index determined from the radial artery pulse under rest conditions (~ −0.8), while *RI* was 6.2 m/s.

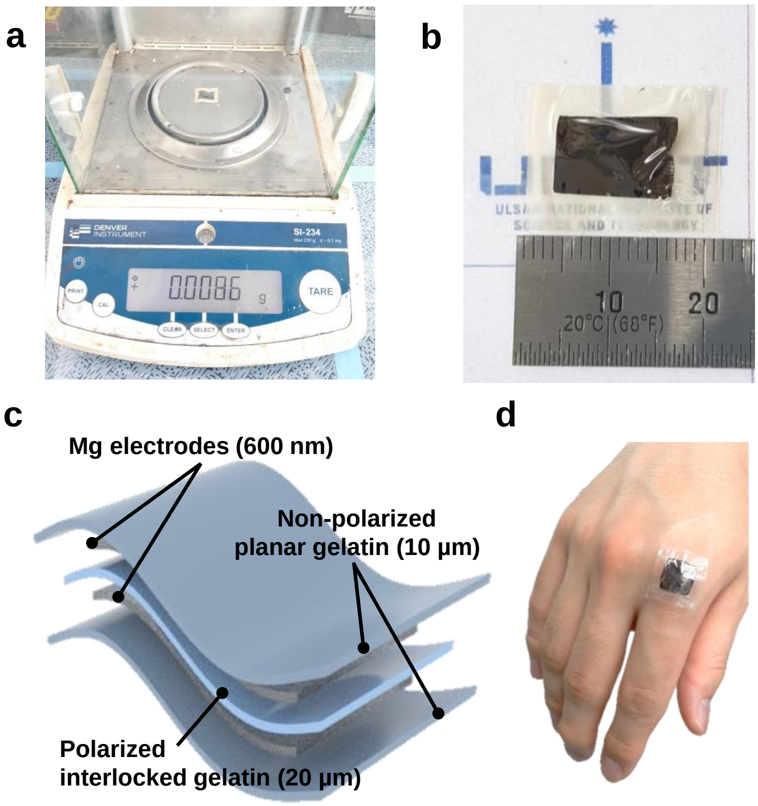

Figure S1. (a) Measurement of the weight and (b) size of the Mg-electrode sputtered microdome pattern gelatine film. (c) Schematic of the device configuration of the gelatine-based interlocked e-skin nanogenerator. (d) Conformable attachment of the e-skin on the curvilinear surface of the human body.

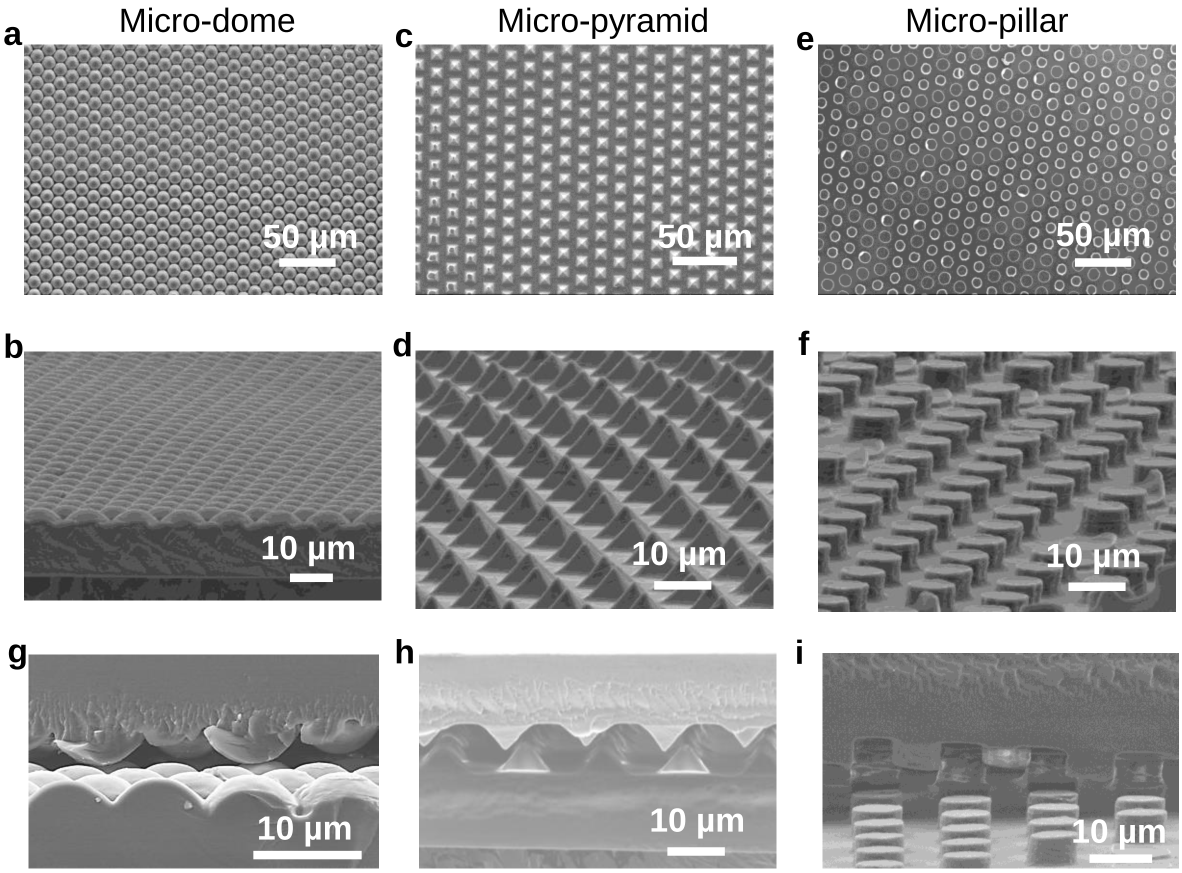

Figure S2. SEM images of the (a,c,e) top view and (b,d,f) tilted view of (a,b) microdome, (c,d) micropyramid, and (e,f) micropillar patterned gelatine films. Cross-sectional SEM images of the interlocked (g) microdome, (h) micropyramid, and (i) micropillar patterned gelatine films.

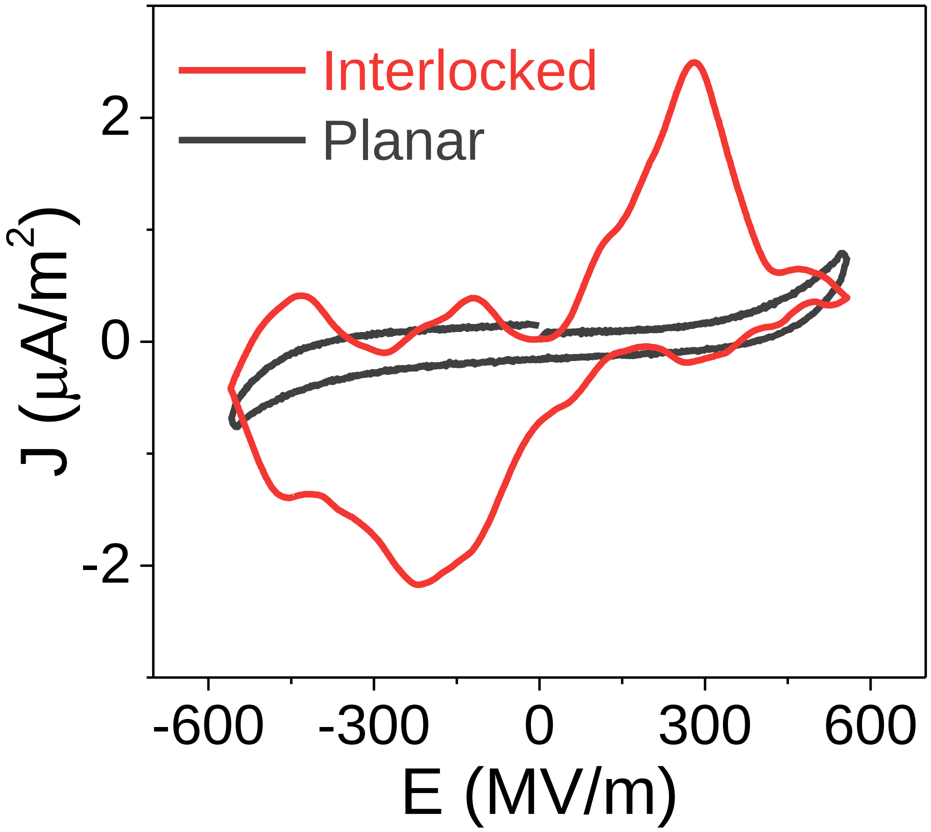

Figure S3. Current density (J) vs. electric field (E) hysteresis loop of the interlocked microdome and planar gelatine films.

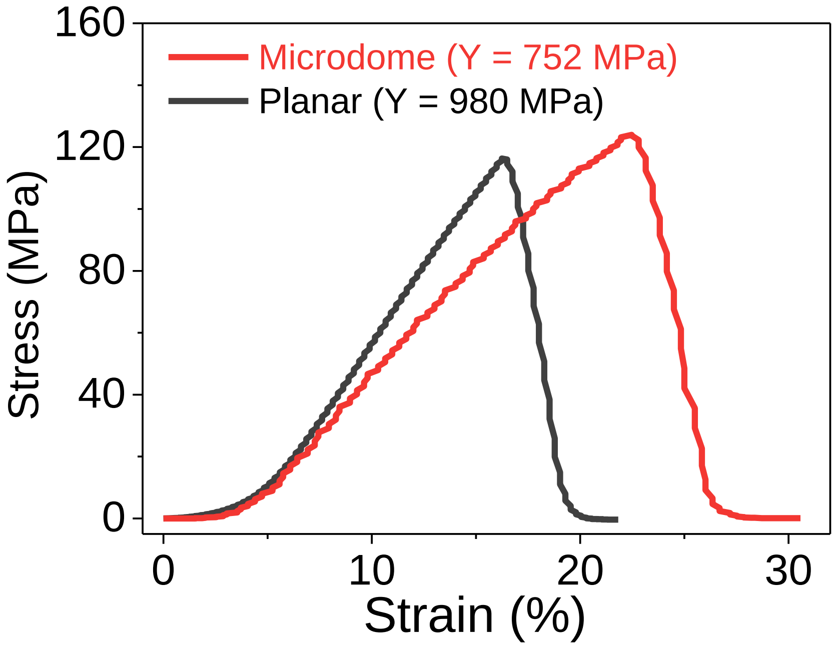

Figure S4. (a) Tensile stress–strain curves of the planar and microdome patterned gelatine films used to evaluate the Young's modulus (Y).

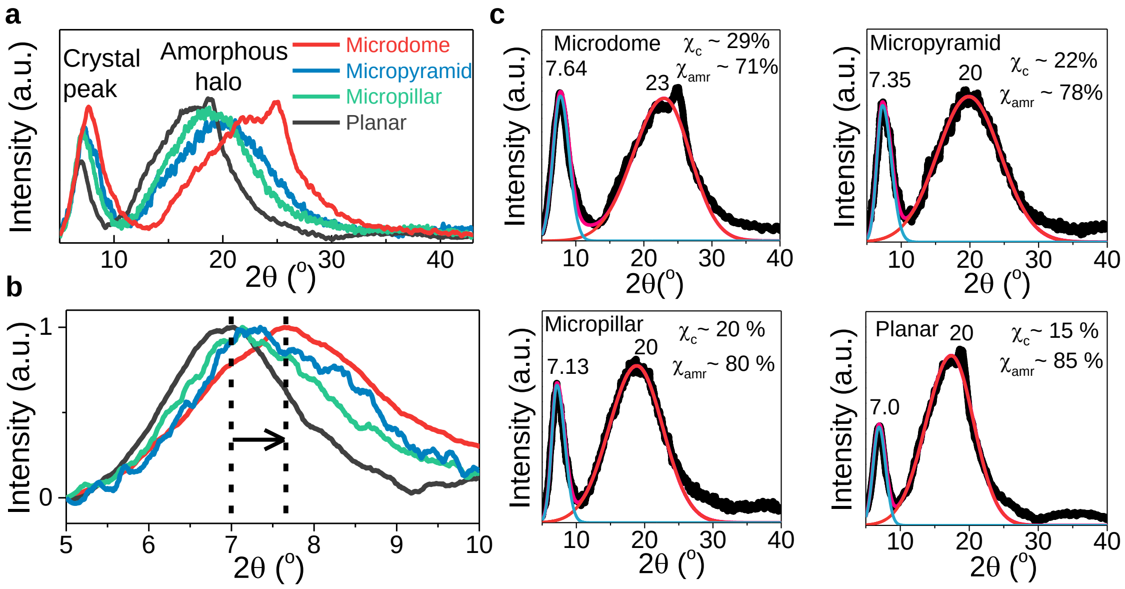

Figure S5. (a) XRD patterns of the gelatine films with different microstructures. (b) Enlarged view of the crystalline peak showing the shift in the crystalline peak to higher angles (e.g., from 2θ ~7° for the planar gelatine film, to 2θ ~7.13° for the micropillar, 2θ ~7.35° for the micropyramid, and 2θ ~7.64° for the microdome gelatine films). As a result, the intermolecular lateral packing spacing (*d*) was reduced from *d* ~1.26 nm for the planar gelatine film to *d* ~1.24 nm for the micropillar, *d* ~1.20 nm for the micropyramid, and *d* ~1.15 nm for the microdome patterned gelatine films. Here, the *d* spacing was calculated using Bragg's equation: $d = \frac{\lambda}{2sin\theta}$, where λ =0.154 nm and the angle θ is in radians. (c) The curve deconvolution technique facilitates the quantification of the total degree of crystallinity (χ_c) of the gelatine films. Here, $\chi_c = \frac{\Sigma A_{cr}}{\Sigma A_{cr}+\Sigma A_{amr}} \times 100\ \%$ where, ΣA_{cr} and ΣA_{amr} are the sums of the integral areas of the crystalline peaks and the amorphous halo from the gelatine films, respectively. χ_{amr} is the amorphous counterpart of the films.

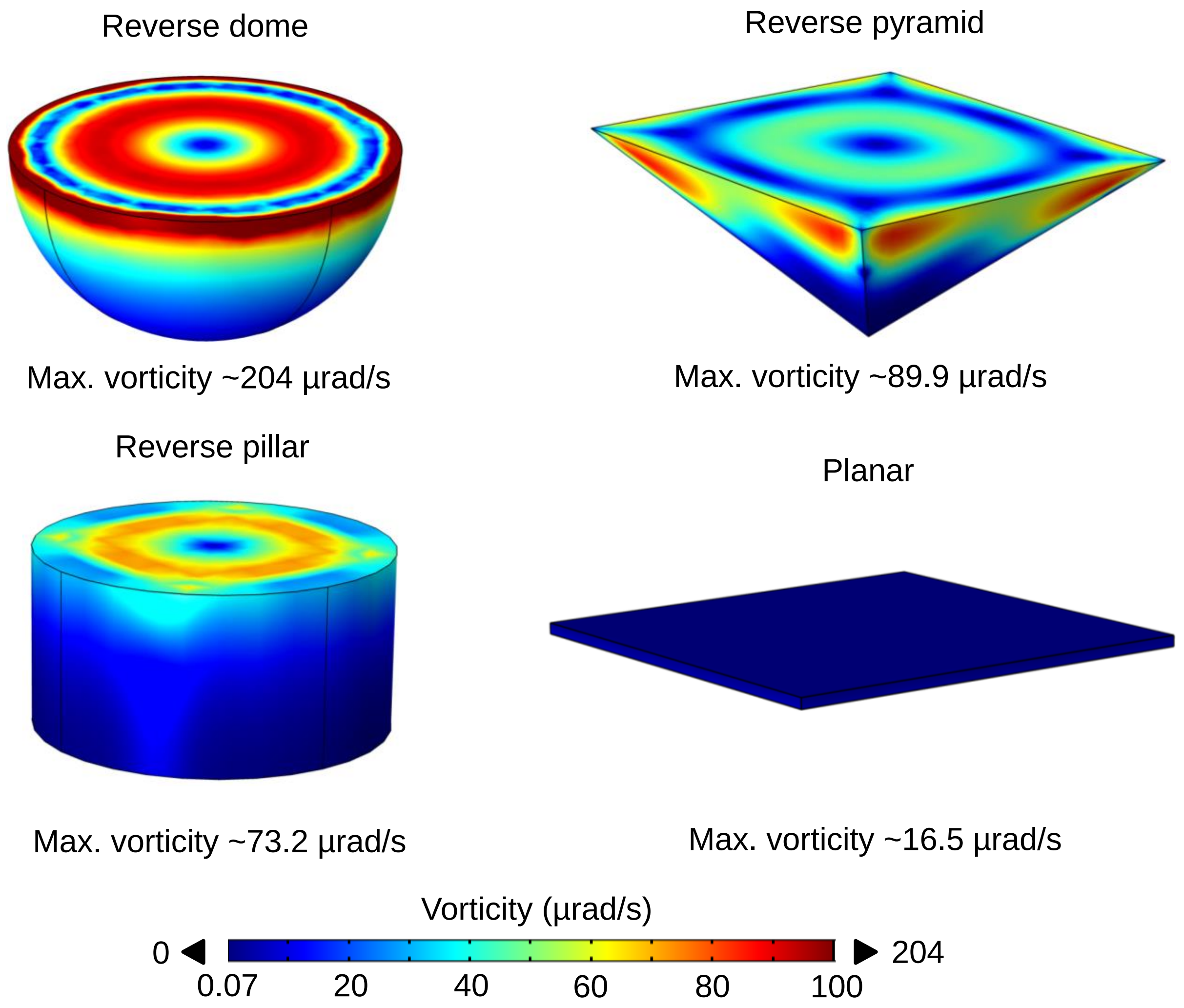

Figure S6. Simulated vorticity distribution of the aqueous gelatine spinning solution within single reverse dome, pyramid, and pillar microstructures, as well as the planar substrate under rotation at 1000 rpm (as for the experimental samples).

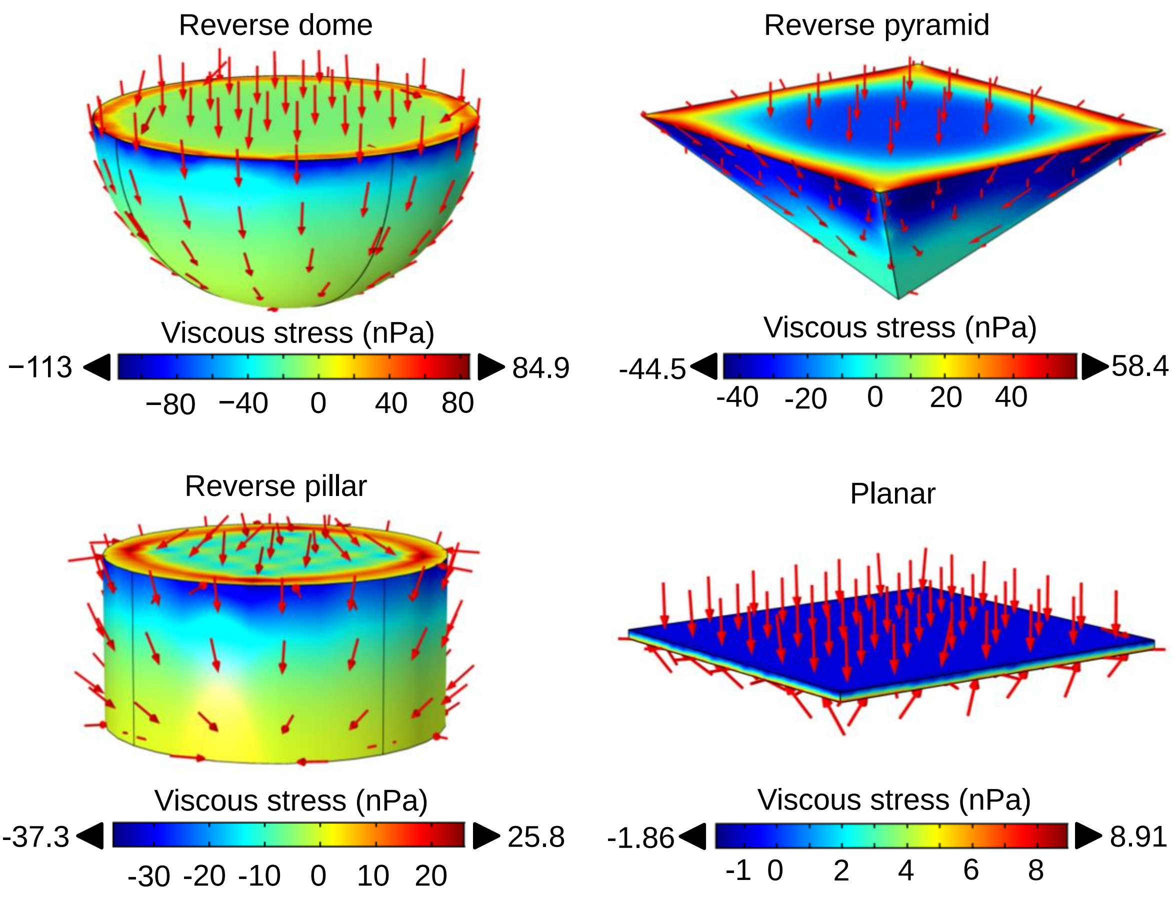

Figure S7. Simulated viscous stress distribution of the aqueous gelatine spinning solution within single reverse dome, pyramid, and pillar microstructures, as well as the planar substrate under rotation at 1000 rpm (as for the experimental samples). The direction of viscous stress is represented by the red arrows, where the bottom side of the reverse pattern substrate corresponds to the top side of the micro-patterned gelatine films.

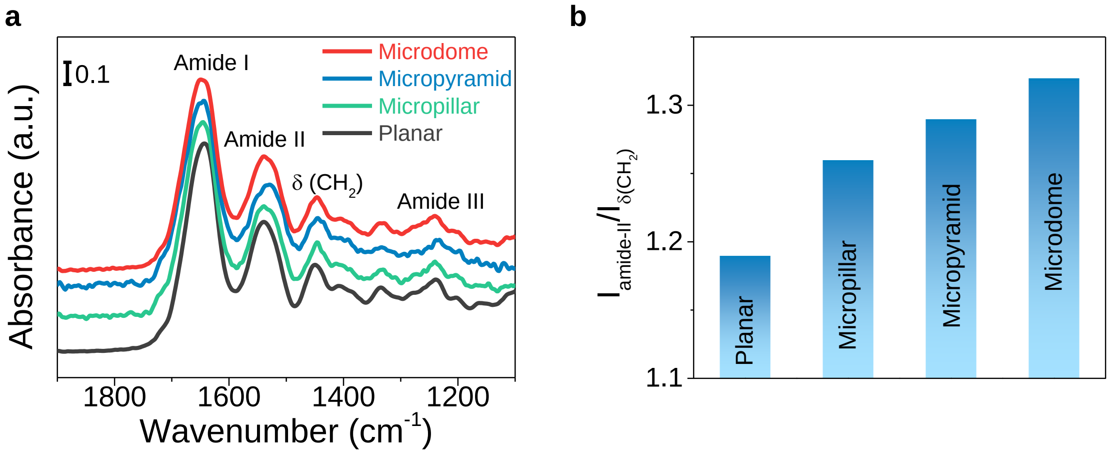

Figure S8. (a) FT-IR spectra and (b) absorption intensity ratio of amide II to δ(CH_2) bands of different microstructured gelatine films.

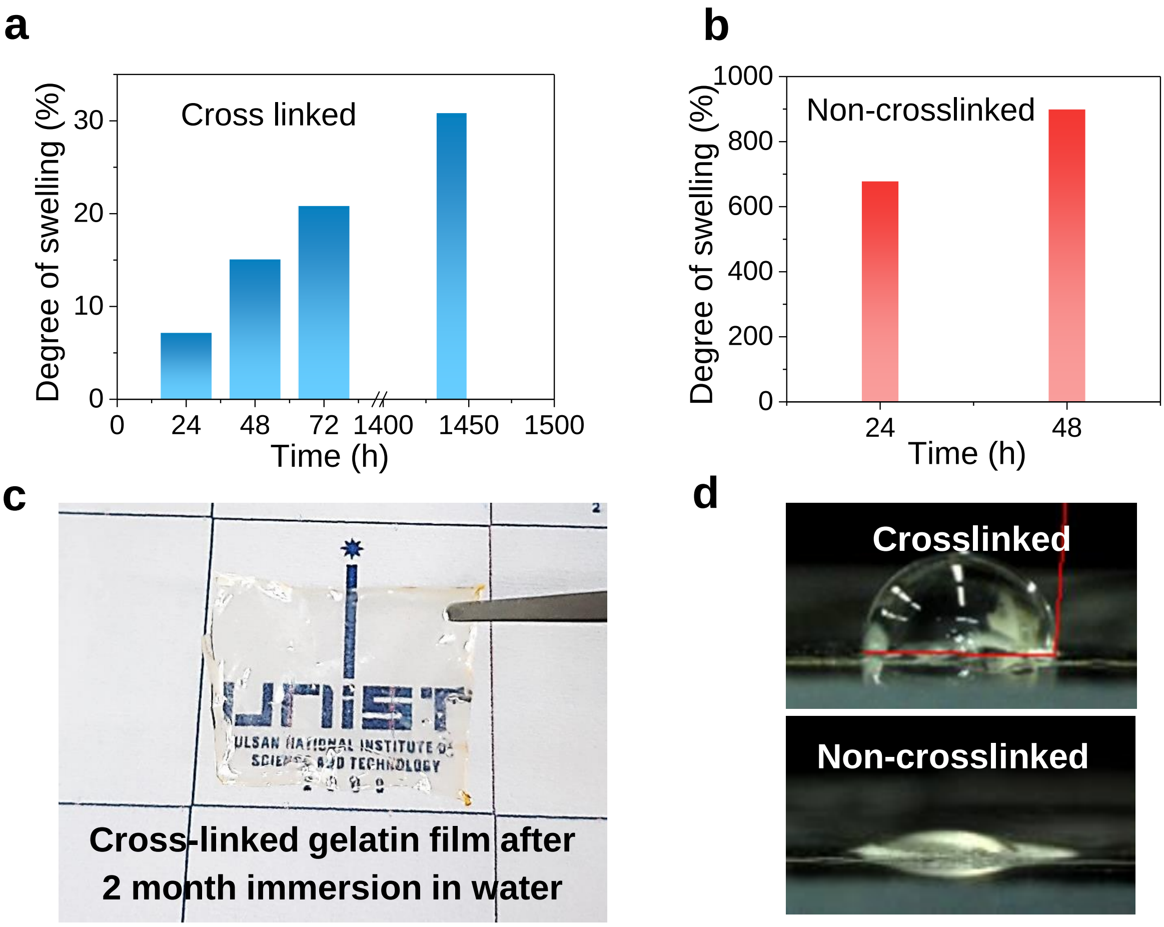

Figure S9. Degree of swelling (%) for (a) crosslinked and (b) non-crosslinked gelatine films. Degree of swelling (%) = $[(w\text{-}w_o)/w_o]\times 100$ %, where w and w_o are wet (after immersion in water) and the initial weights of the sample, respectively. (c) Photograph of the crosslinked gelatine film after 2 months immersion in water, which retained its original shape and size. Photographs used to determine the contact angle of (d) crosslinked (~92.97^o) and (f) non-crosslinked (~0°) gelatine films

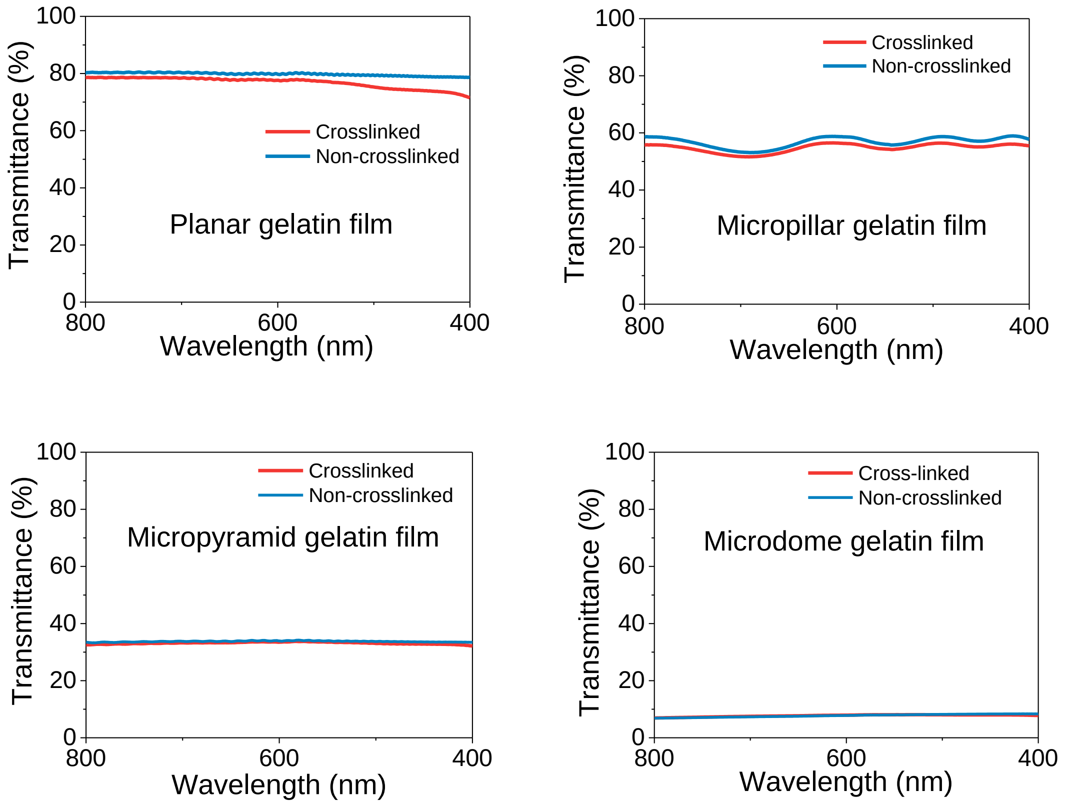

Figure S10. Transmittance of the microstructured gelatine films within the visible wavelength (800–400 nm) before and after crosslinking with glutaraldehyde.

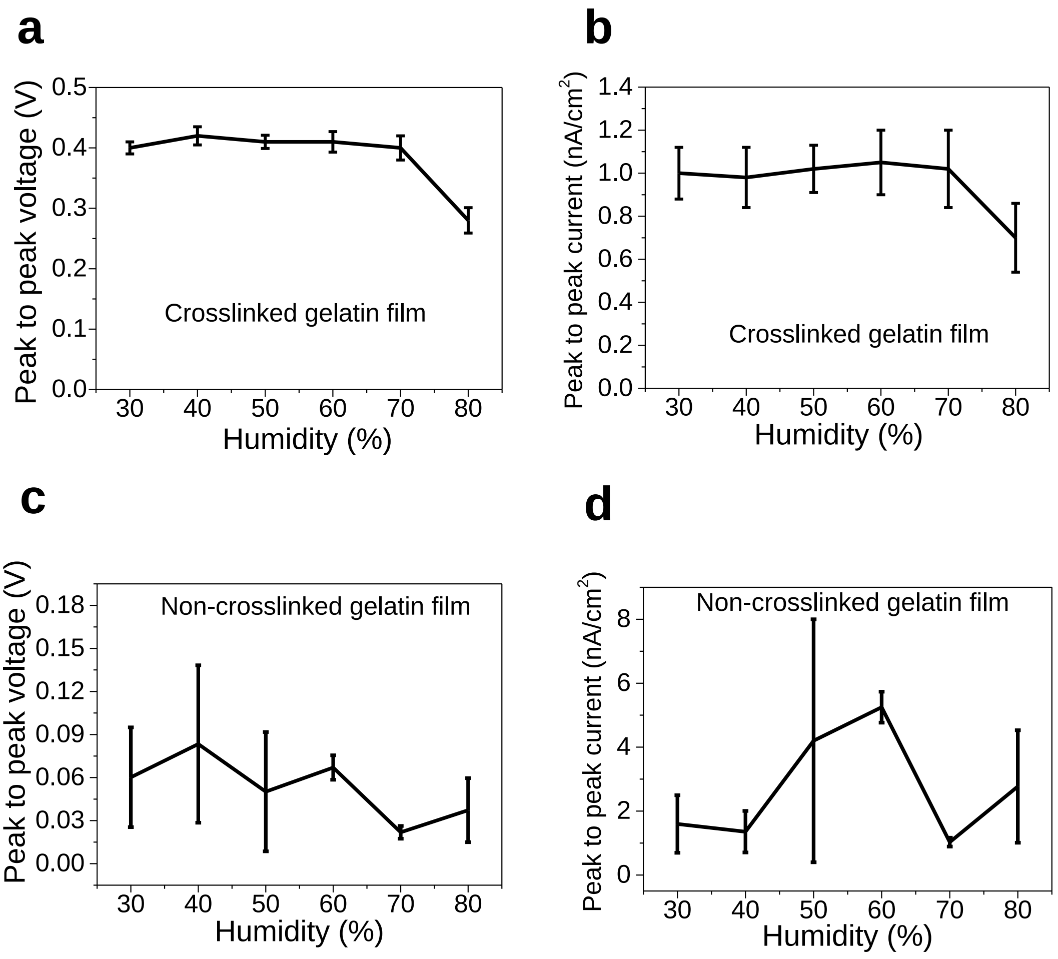

Figure S11. Humidity dependent (30−80%) (a,c) peak-to-peak output voltage and (b,d) peak-to-peak output current of (a,b) crosslinked and (c,d) non-crosslinked gelatine films. The slight decreased in the output performance of the crosslinked planar gelatine film at 80% humidity was due to the gelatine film absorbing a large amount of moisture at high humidity, which induced crumpling of the film during crosslinking. Therefore, all other devices were fabricated below 70% humidity, typically at 40% humidity.

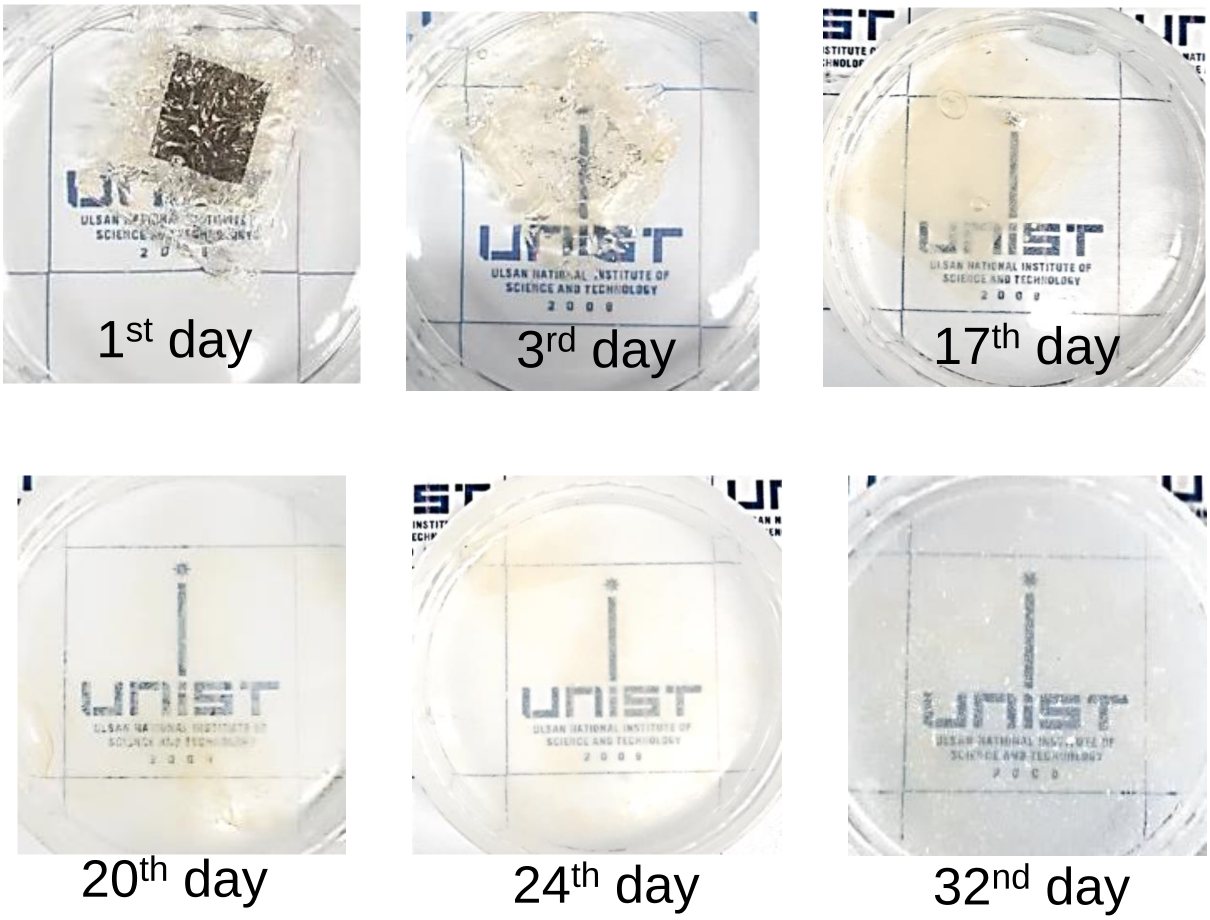

Figure S12. Photographs of the biodegradation of an interlocked microdome gelatin film based device.

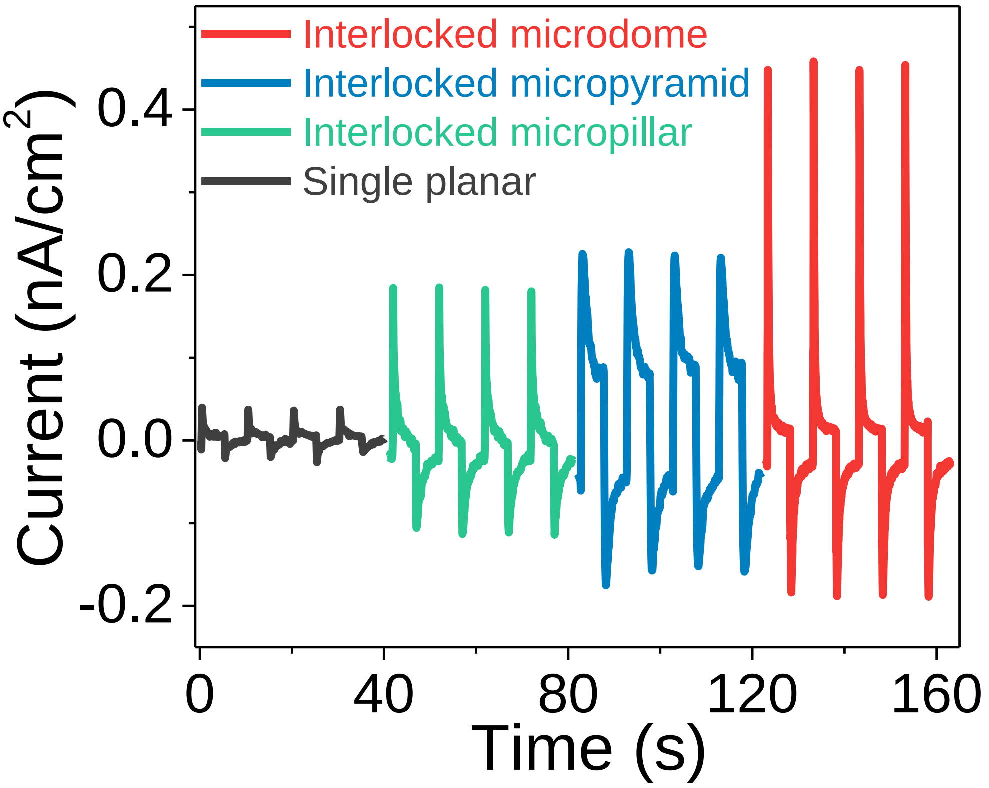

Figure S13. Comparison of the pyroelectric output currents of the microstructured devices under $\Delta T \sim 1.8\ K$.

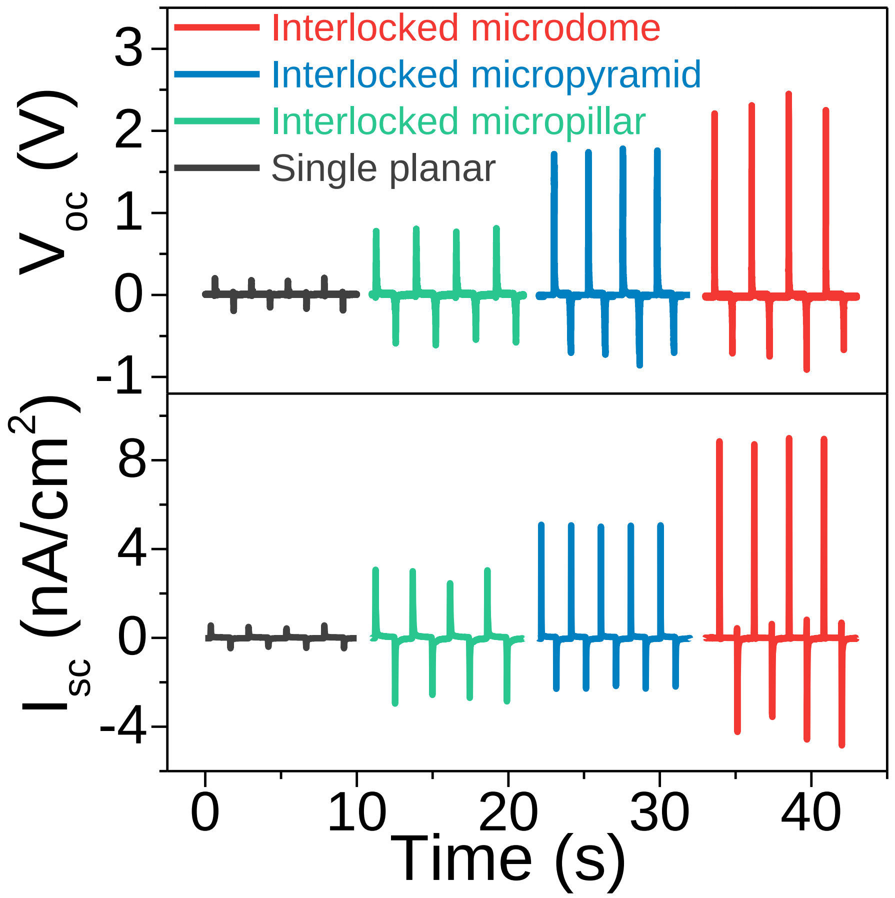

Figure S14. Comparison of the piezoelectric output voltages and currents from the microstructured devices subjected to σ ~113 kPa.

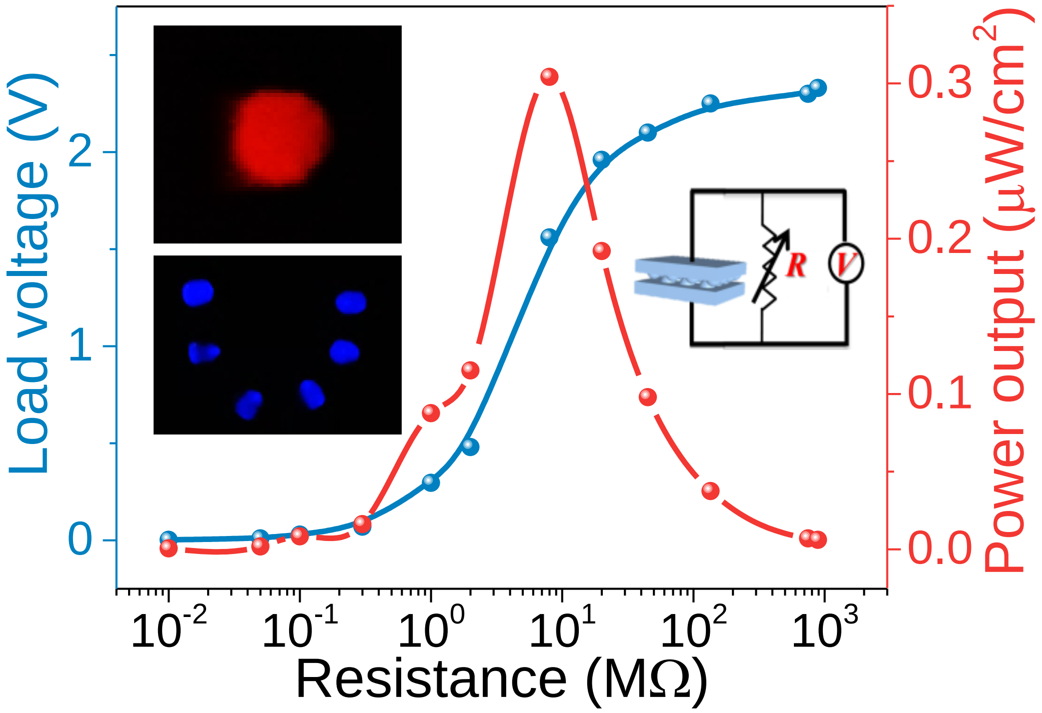

Figure S15. Output voltage and power density from an interlocked microdome energy harvester measured across the load resistors using the electrical circuit (right inset), which operated an array of 6 blue LEDs and one red LED (left inset). The maximum achieved power density was 0.3 μW/cm^2 with an external load resistance of 8 MΩ.

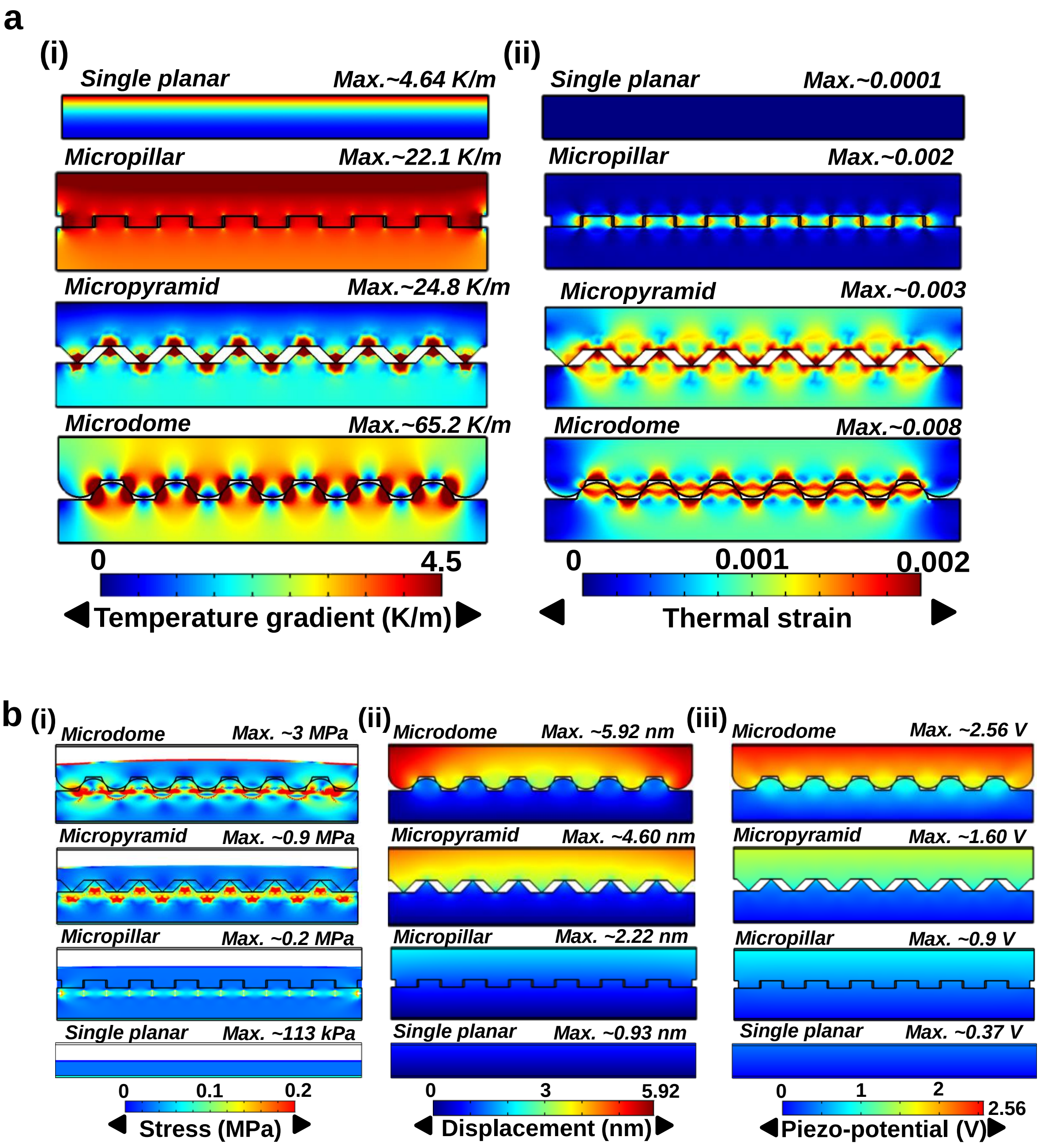

Figure S16. (a) Results of the FEM-based simulation under the experimentally obtained thermal pulse of ΔT ~ 1.8 K for (i) temperature gradient confinement and (ii) thermal strain distribution generated within the devices. (b) Results of the FEM-based simulation under 113 kPa vertical pressure for (i) stress, (ii) displacement, and (iii) piezoelectric potential distribution (consistent with the experimental results).

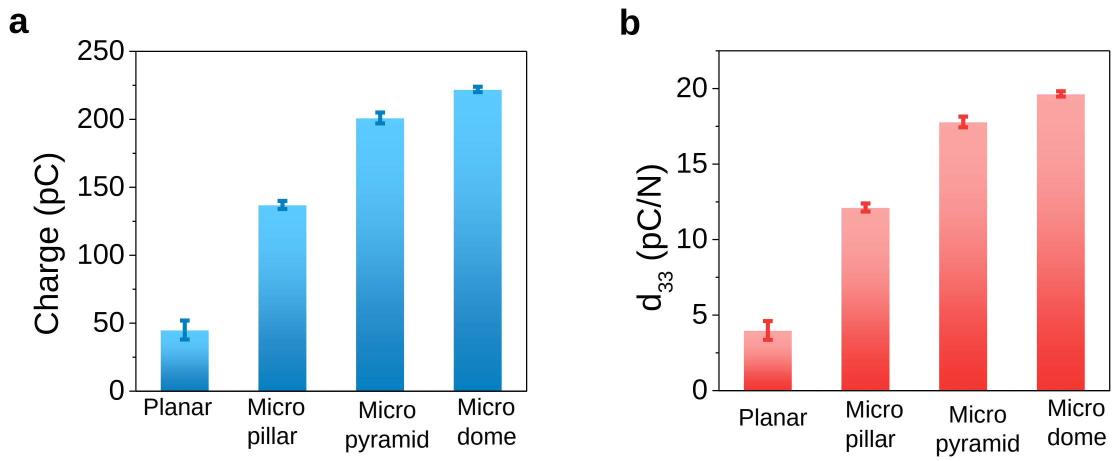

Figure S17. (a) Generated charge under pressure (~113 kPa) and the corresponding (b) d_{eff} values of all device types.

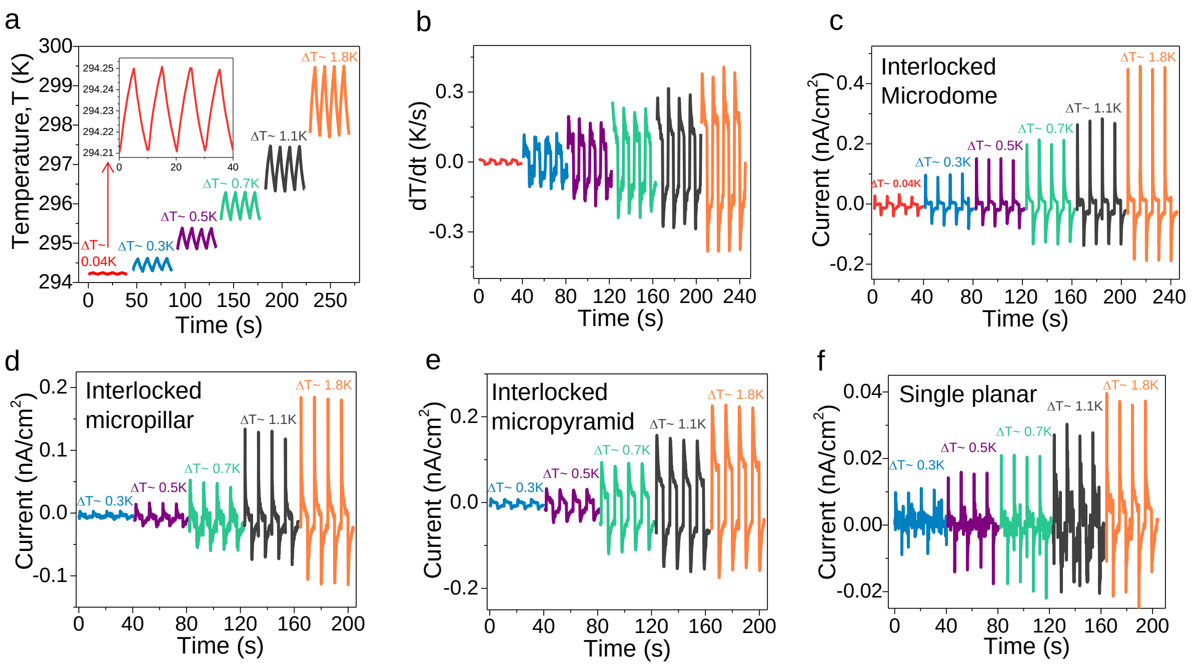

Figure S18. (a) Time-dependent temperature profile for ΔT = 0.04–1.8 K and the corresponding (b) dT/dt = 0.01–0.4 K/s applied to the devices that generated the time-dependent output current from the interlocked (c) microdome, (d) micropillar, and (e) micropyramid structured devices, and the (f) planar device.

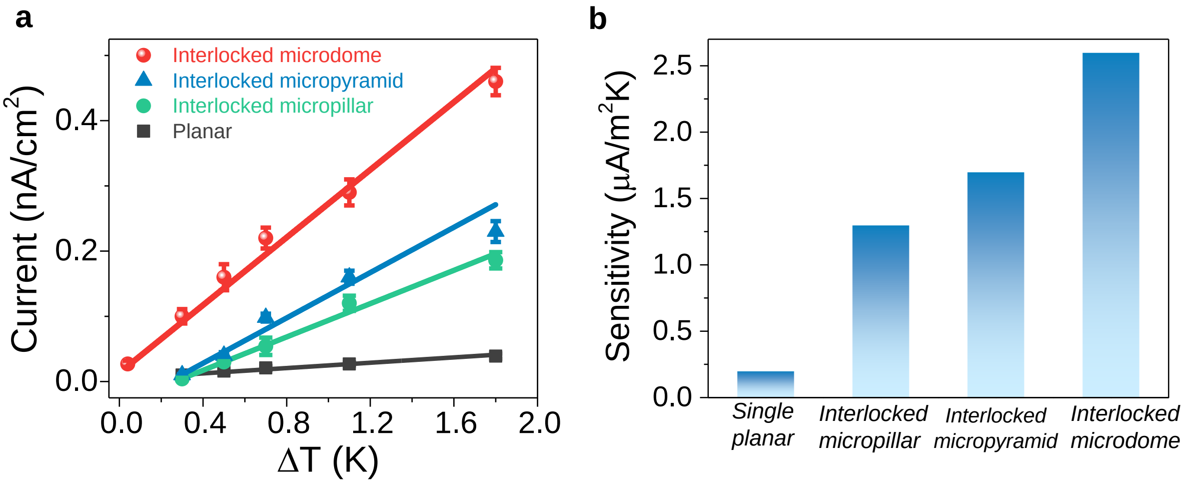

Figure S19. (a) ΔT-dependent output current and (b) thermal sensitivity of the different microstructured gelatin-based e-skins calculated from the slope of the curves in (a).

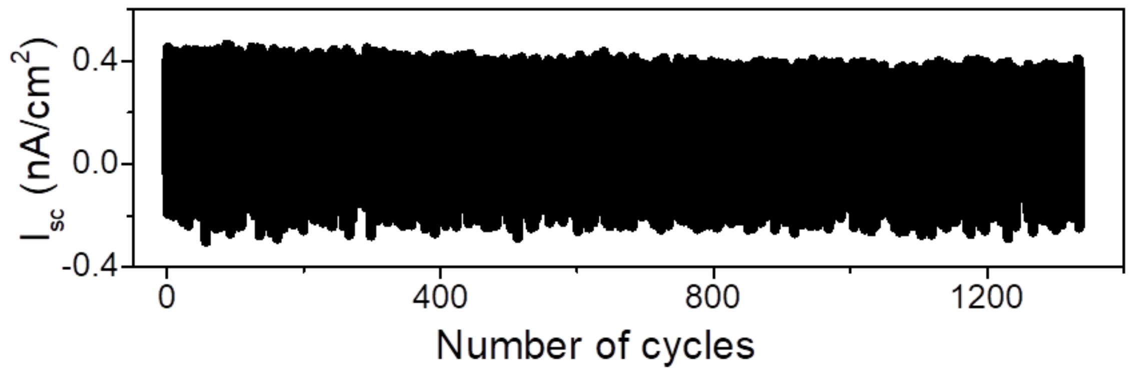

Figure S20. Stable pyroelectric output performance of the interlocked microdome device under ΔT ~1.8 K.

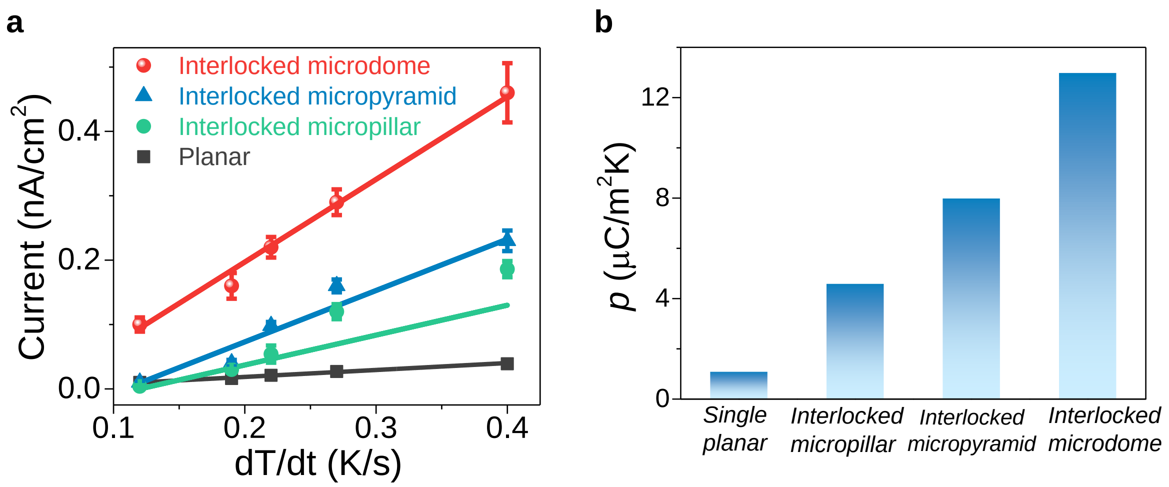

Figure S21. (a) The dT/dt-dependent output current and (b) pyroelectric coefficient of the different microstructured gelatin-based e-skins calculated from the slope of the curves in (a).

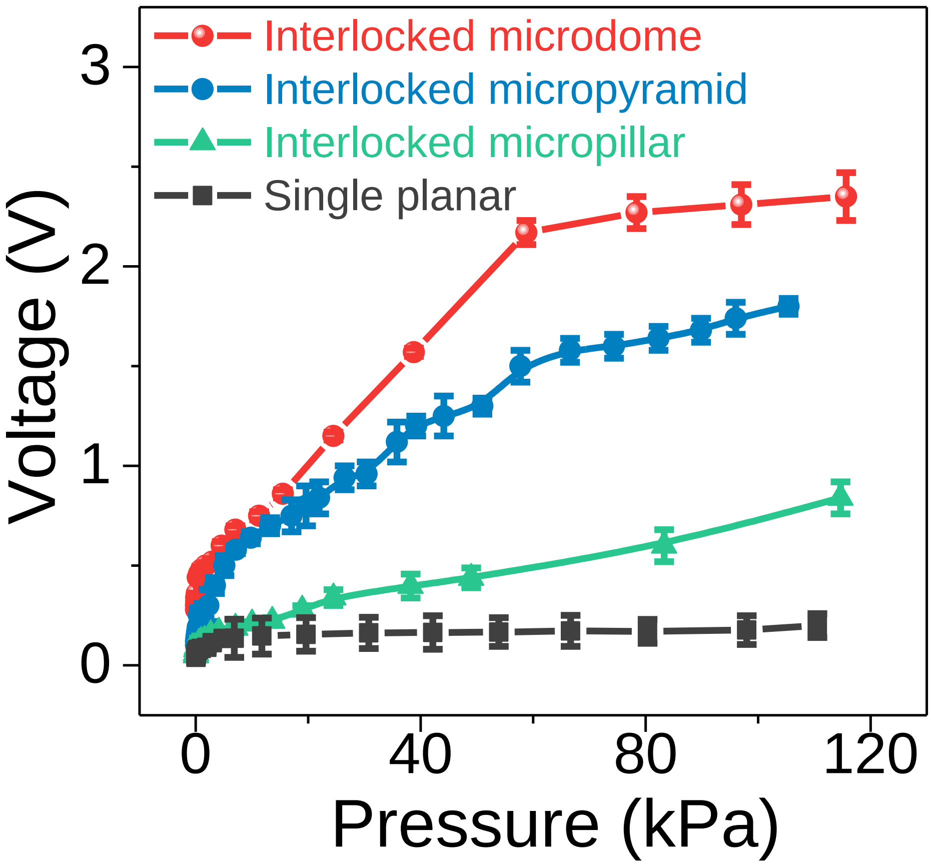

Figure S22. Output voltage from the different interlocked structures and planar gelatine film devices measured over the pressure range of 40 Pa to 113 kPa.

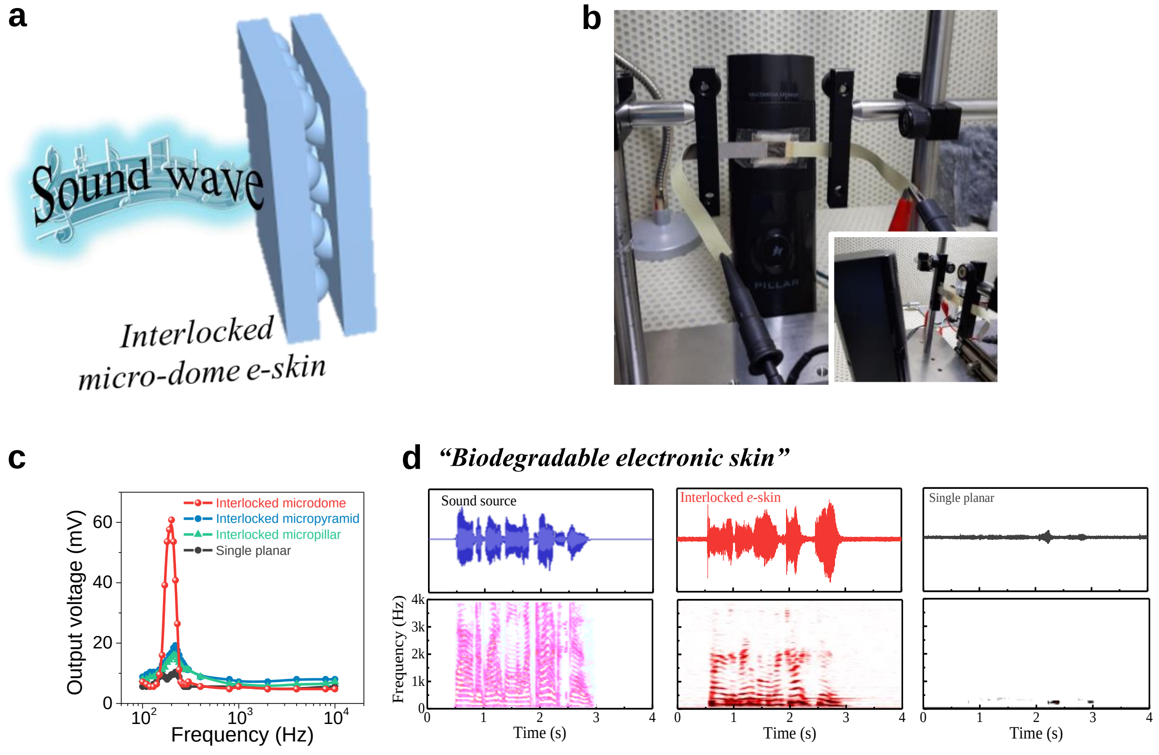

Figure S23. (a) Schematic of the interlocked microdome e-skin detecting an acoustic sound wave. (b) Measurement setup with the *e*-skin placed 2.5 cm away from the audio speaker. (c) Output voltage from *e*-skins as a function of sound frequency (100 Hz to 10 kHz) at an average sound pressure level of 97 dB. (d) Demonstration of the excellent responsivity to an external waveform (acoustic recording of "*Biodegradable electronic skin*") by an interlocked microdome e-skin and its corresponding STFT spectrogram compared with the poor response of the single planar structured device.

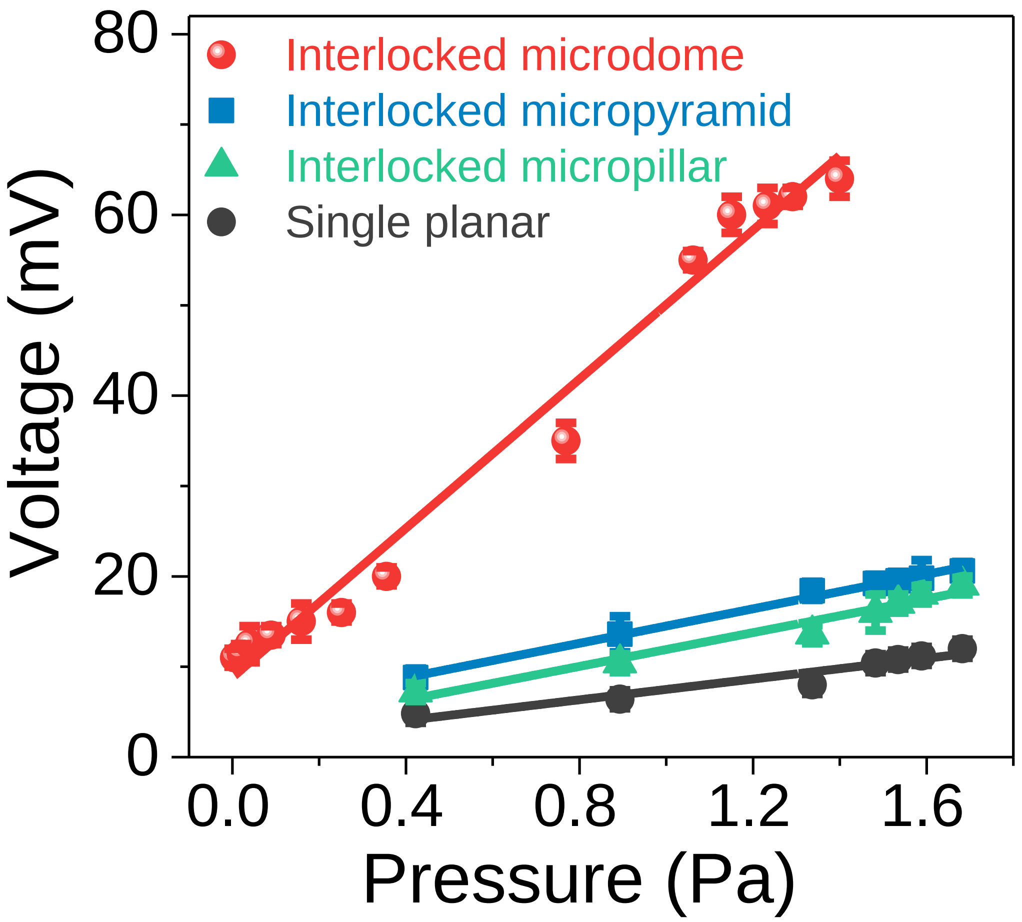

Figure S24. Pressure-dependent output voltage over the ultralow pressure region (< 2 Pa) for all devices.

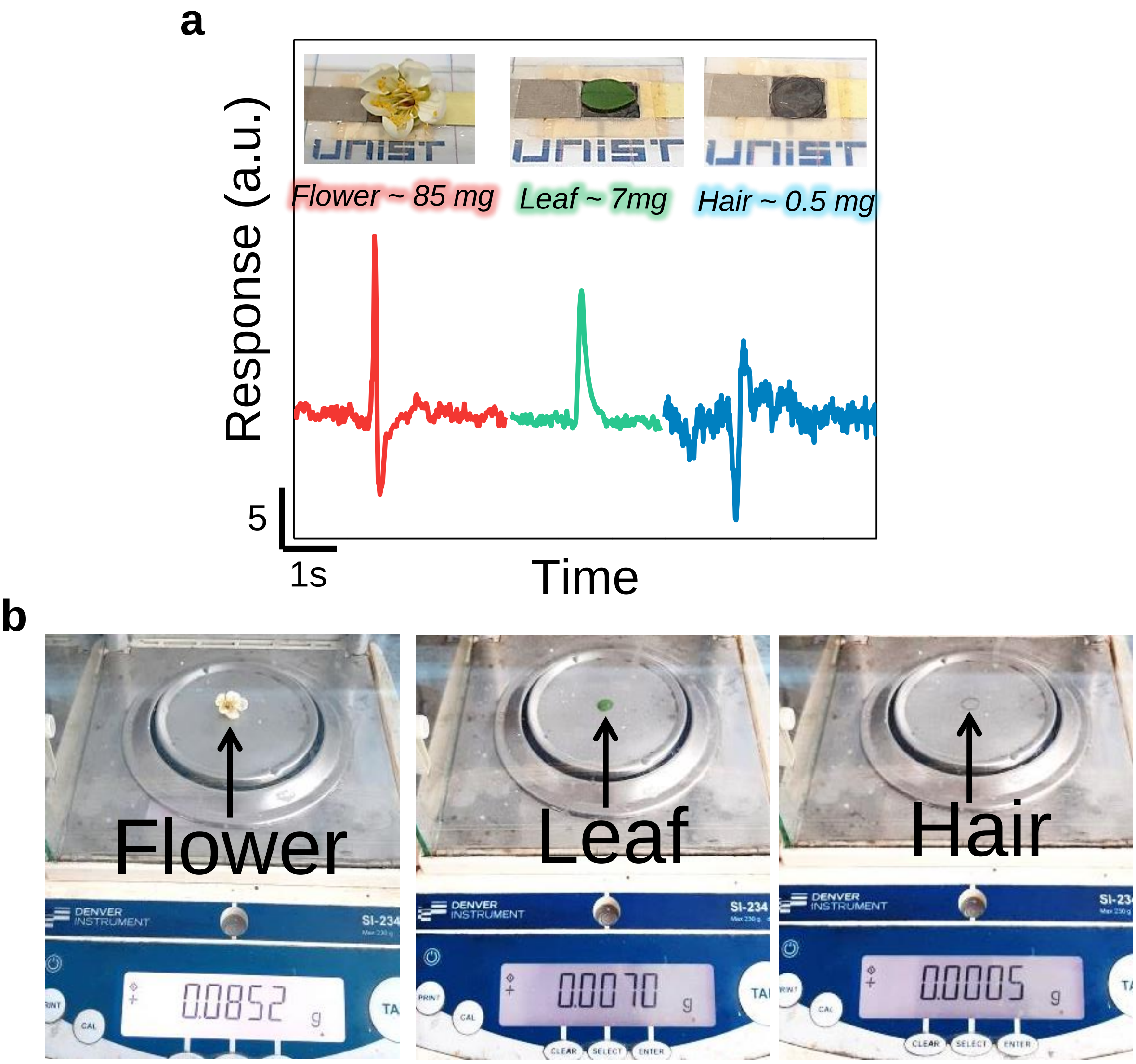

Figure S25. (a) Output responses from the microdome patterned device for detecting the (b) small weights of a flower, leaf, and hair, demonstrating the excellent pressure-sensing capability of the e-skin.

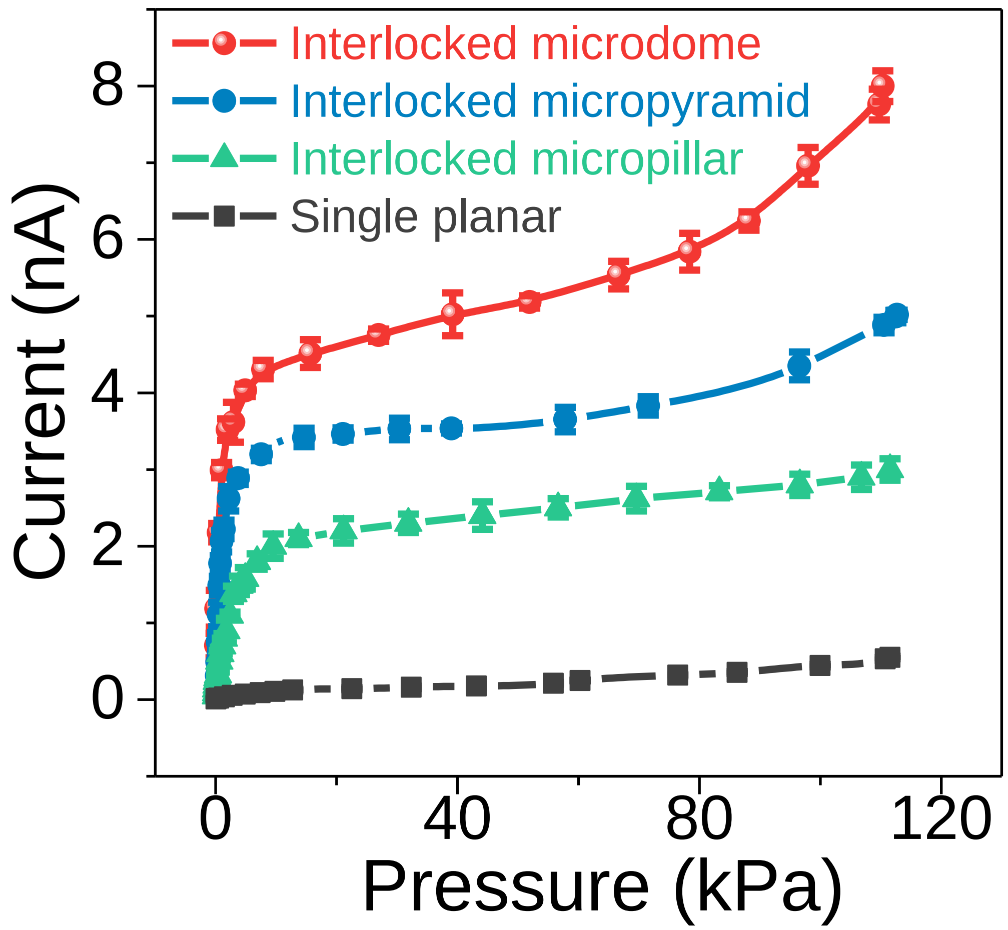

Figure S26. Pressure-dependent output current from all devices for the pressure range of 40 Pa to 113 kPa.

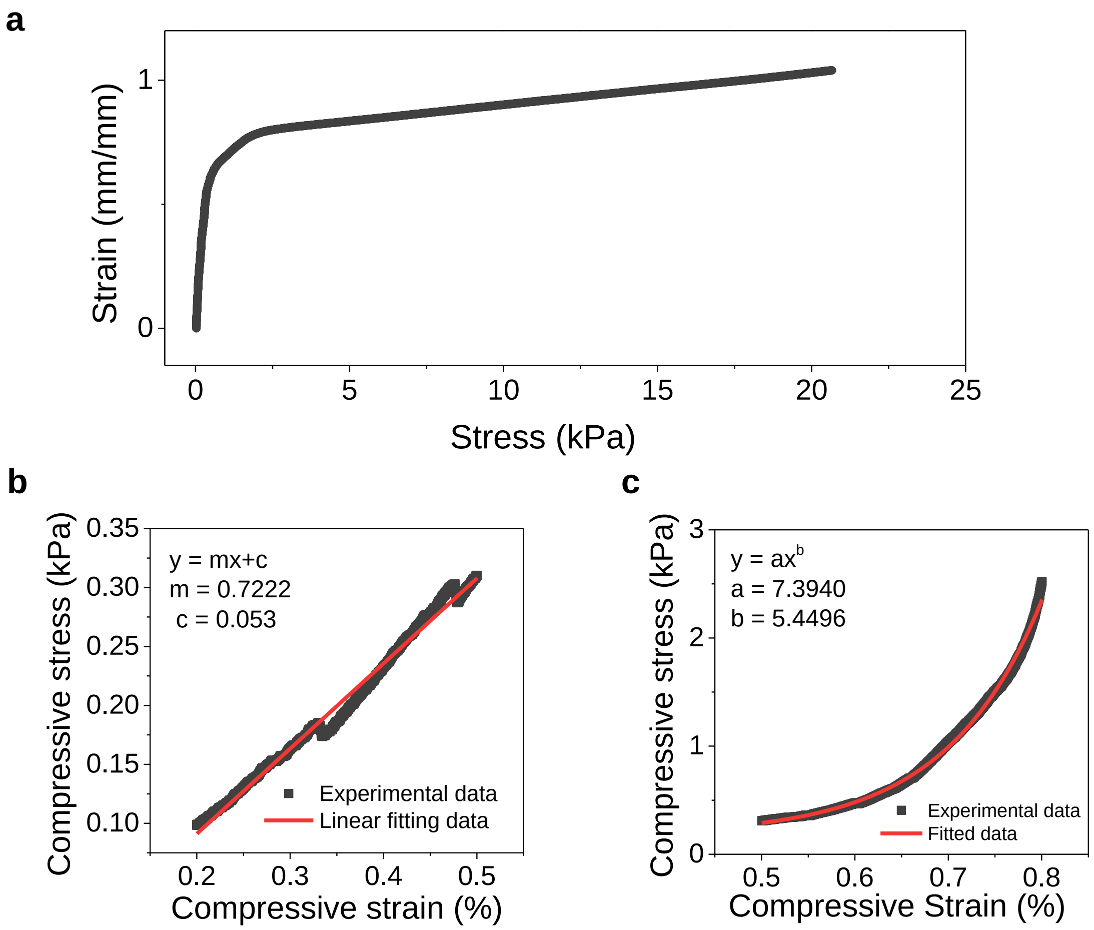

Figure S27. (a) Compressive stress (σ)−strain (ε) curves of the interlocked microdome device. Compressive σ–ε curves in the range of (b) 0−0.5% strain with linear fitting and (c) 0.5−0.8% strain with their power law fits. The equations used for fitting are shown in the respective graphs, which yielded $\sigma\ (kPa) = 0.72\varepsilon$ for low strain (0.2–0.5%) and $\sigma\ (kPa) = 7.4\varepsilon^{5.4}$ for higher strain (0.5–0.5%).

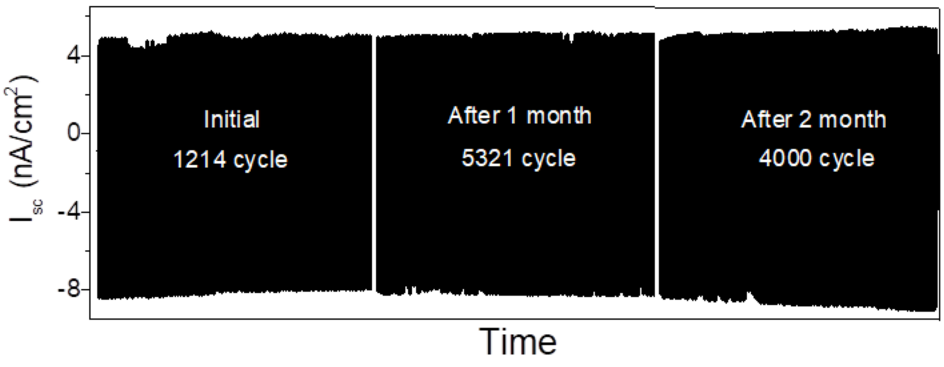

Figure S28. Stability of the piezoelectric output performance of the interlocked microdome patterned e-skin under 113 kPa of pressure.

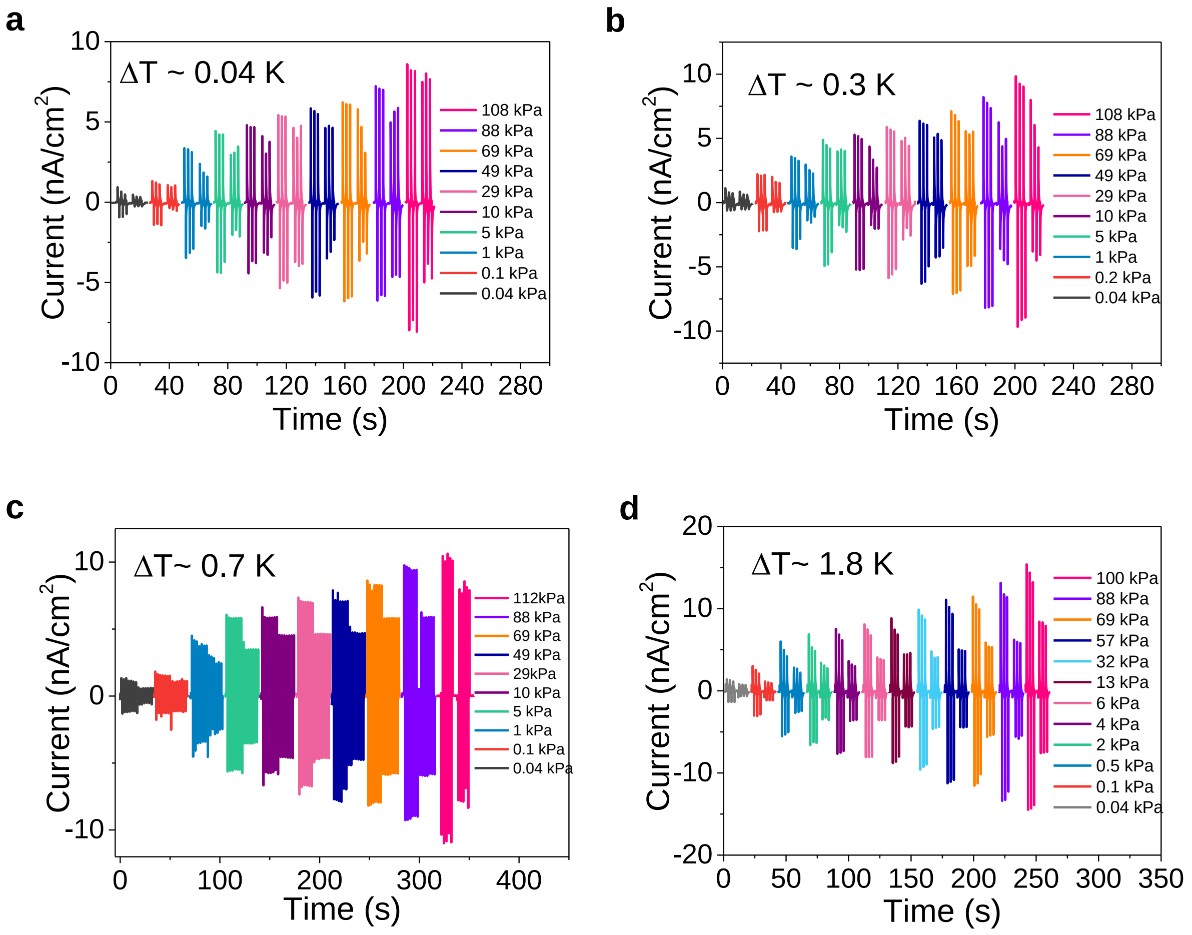

Figure S29. The pyroelectric coupled pressure-dependent piezoelectric output response over the subtle to medium pressure range under applied ΔT of (a) 0.04 K, (b) 0.3 K, (c) 0.7 K, and (d) 1.8 K.

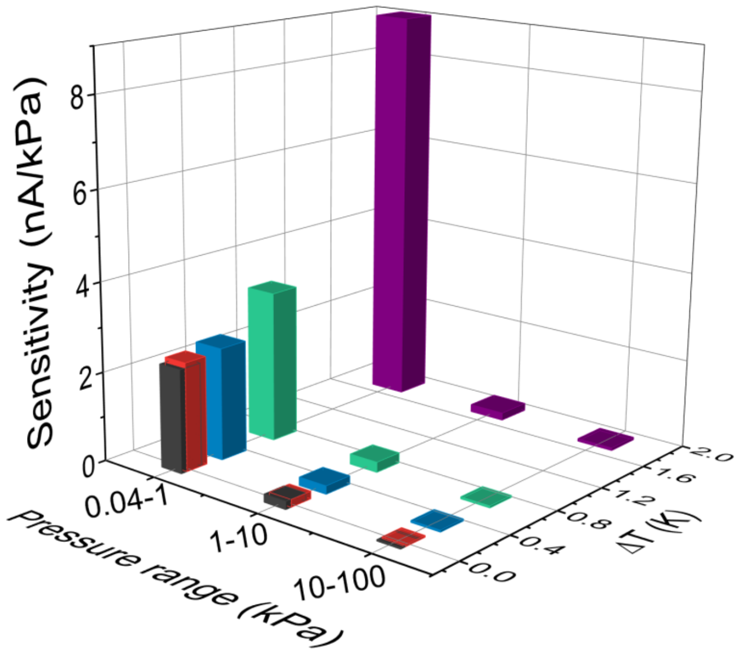

Figure S30. The overall sensitivity of the interlocked e-skin under simultaneous applied pressure and temperature.

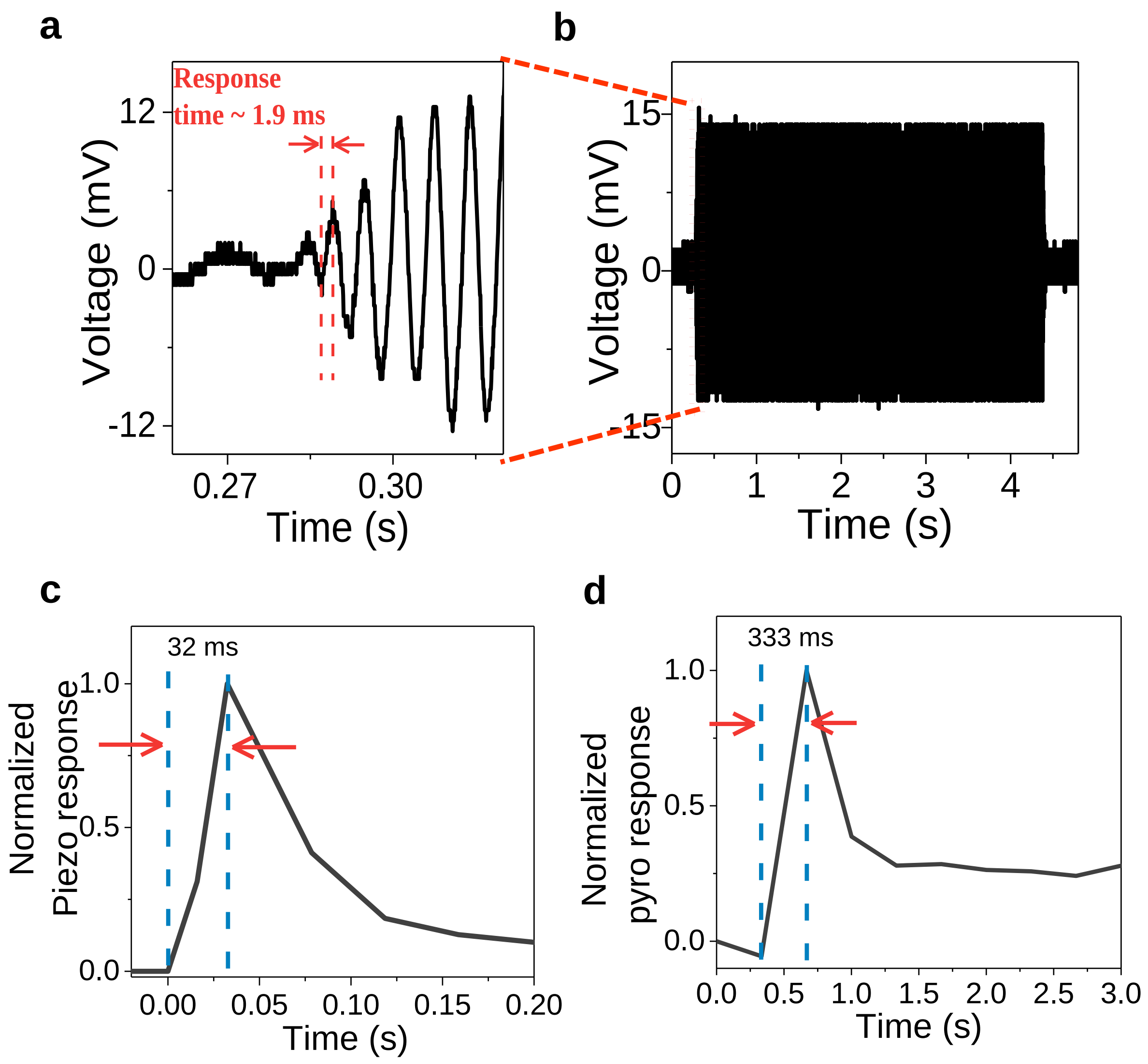

Figure S31. (a) Enlarged view of the (b) time-dependent output voltage showing the response time of an interlocked microdome patterned energy harvester exposed to an acoustic wave with a frequency of 200 Hz and pressure of 0.15 Pa. Response time of the (c) piezoelectric output under 113 kPa and (d) pyroelectric output under ΔT ~0.04 K.

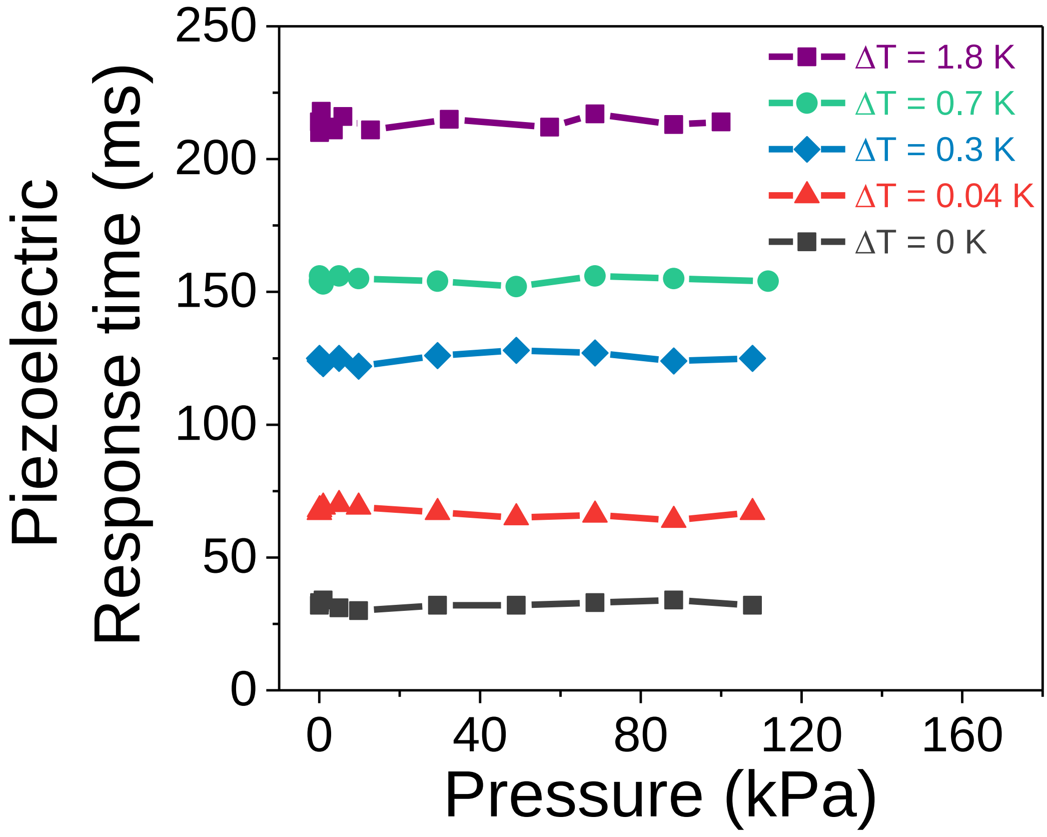

Figure S32. Temperature-dependent variation of the piezoelectric response time, which was almost constant over the pressure range of 40 Pa to 113 kPa for a particular ΔT.

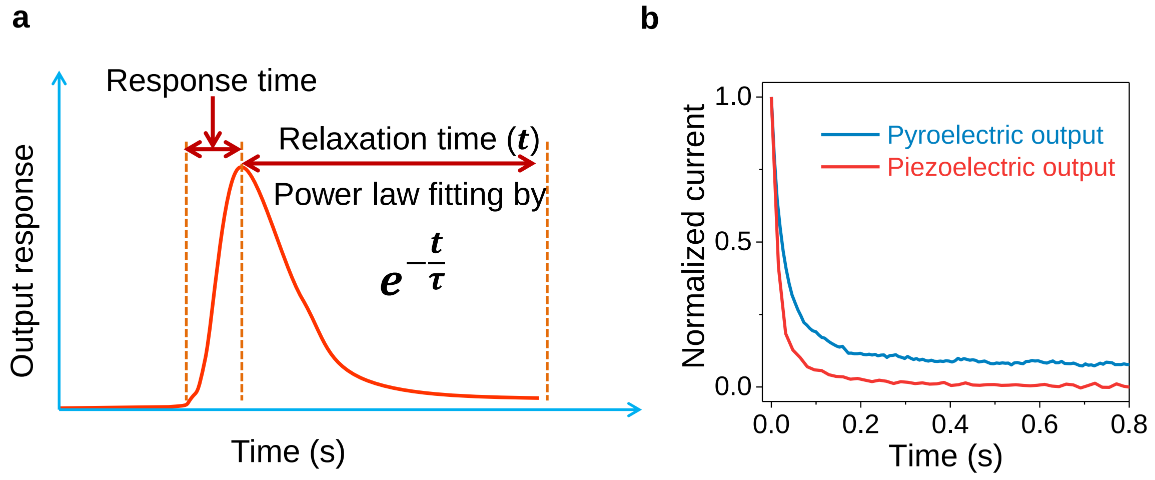

Figure S33. (a) Schematic of a typical output response defining the response time and relaxation time. (b) Representative experimental data for the charge decay of normalised pyroelectric (ΔT ~1.8 K) and piezoelectric (σ ~113 kPa) outputs.

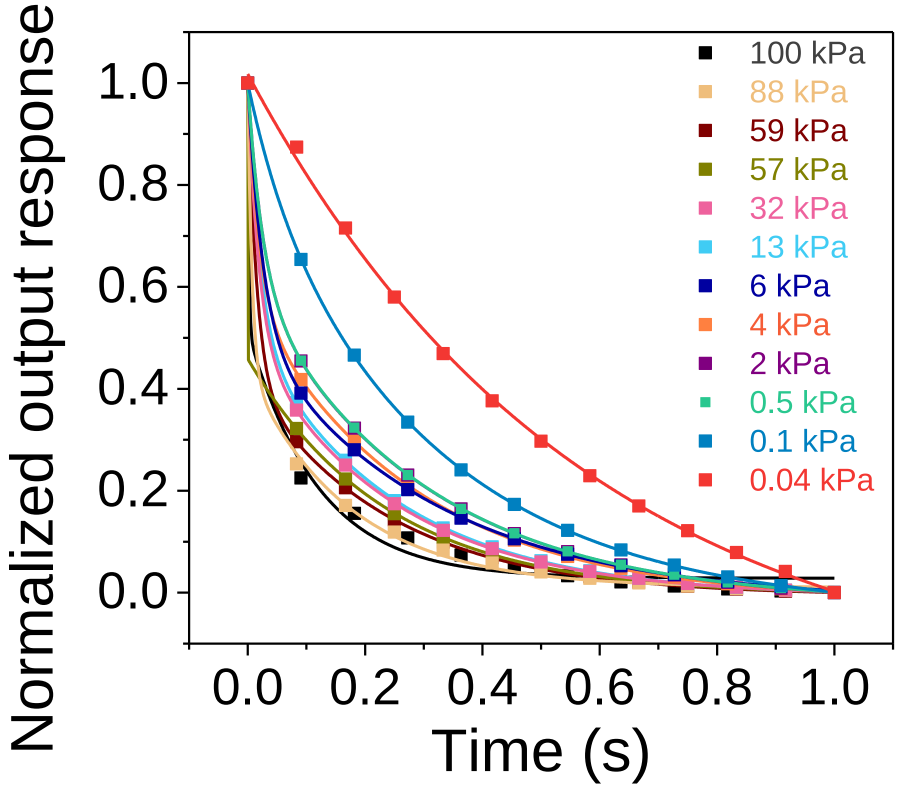

Figure S34. Power law fitting of the normalised piezoelectric charge decay under ΔT ~1.8 K. Longer relaxation times were measured for lower applied pressures.

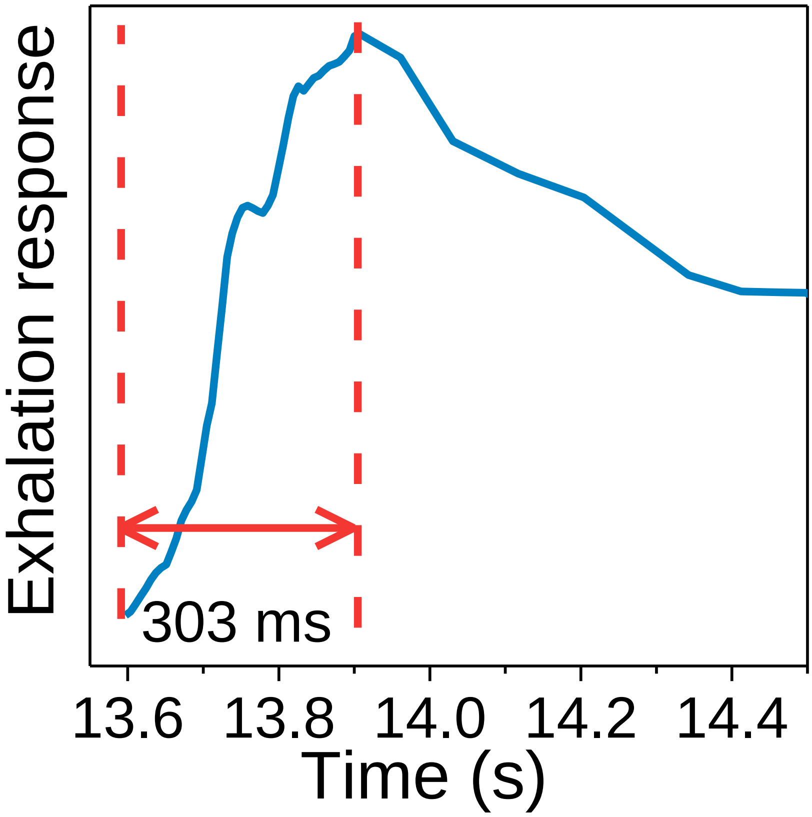

Figure S35. Definition of the exhalation response time (~ 303 ms) which was used with the calibration equation $\tau_R = a\Delta T^b$ to yield the temperature variation during breathing $\Delta T = (\frac{303}{177})^{\frac{1}{0.3}} = 6$ K.

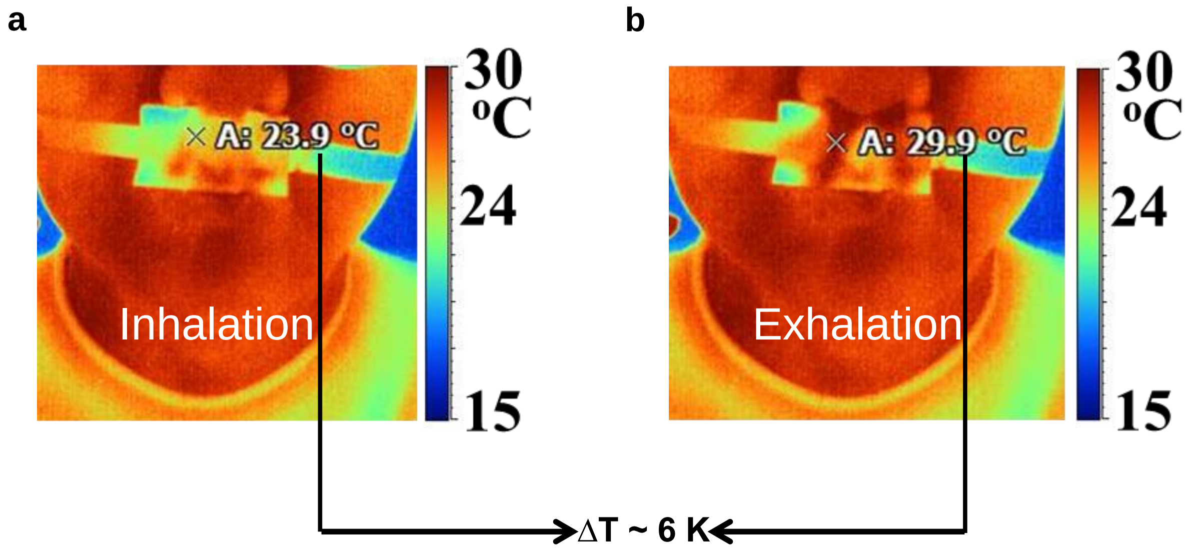

Figure S36. Infrared camera images showing the e-skin temperature during (a) inhalation and (b) exhalation.

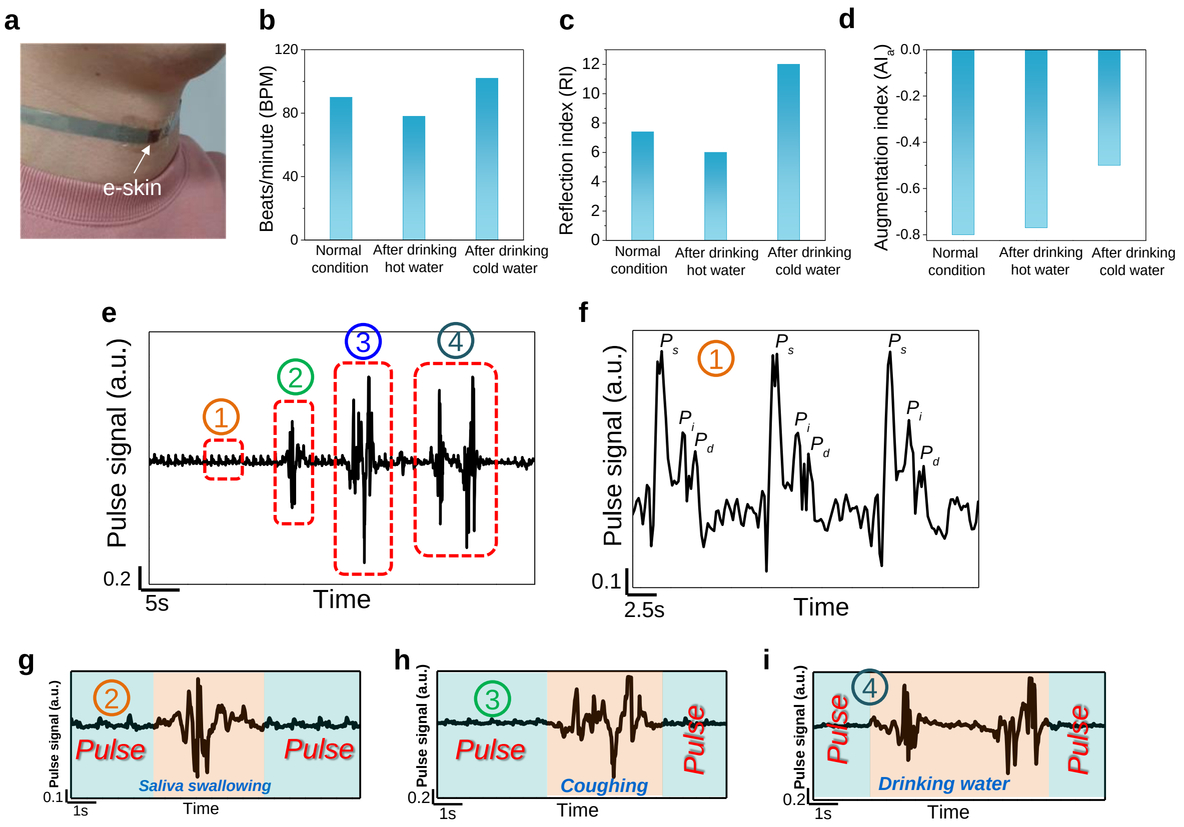

Figure S37. (a) Photograph showing the seamless attachment of the interlocked e-skin to the throat using a spray bandage. Thyroid arterial pulse wave parameters were measured under normal conditions, and after drinking hot or cold water: (b) heart rate, (c) reflection index $RI = \frac{h}{\tau_r}$ (h is the height of the patient and τ_r is the time delay between P_s and P_d waves shown in Fig. 4d in the main manuscript), and (d) augmentation index ($AI_a = \frac{(P_i - P_s)}{(P_s - P_d)}$). The thyroid artery pulse wave (Fig. 4d in the main manuscript) contains various morphological features in the time domain: systolic main wave (P_s), predicrotic wave (P_i), and dicrotic wave (P_d) waves. (e) Thyroid artery pulse signal measurement during various physical activities: (f) rest condition, (g) swallowing saliva, (h) coughing, and (i) drinking water.

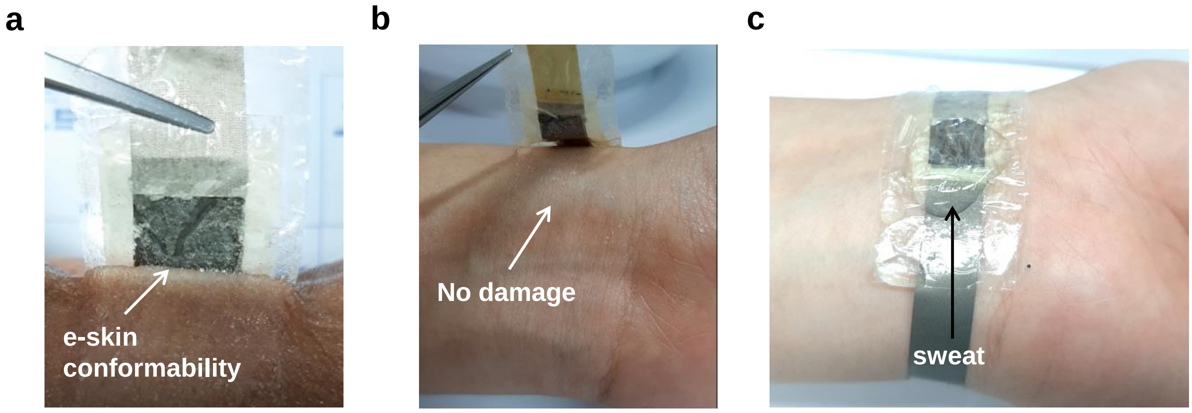

Figure S38. (a) Photographs demonstrating the human skin conformability of our developed interlocked e-skin and the (b) minimal effect of the e-skin on human skin after 6 h of continuous attachment. (c) A droplet of artificial sweat covering the surface area of the *e*-skin indicates its ability to withstand sweating and other humid conditions.

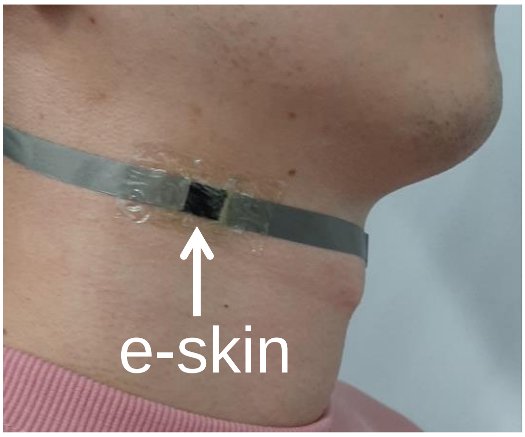

Figure S39. Photograph showing the conformal attachment of the interlocked e-skin to the neck of a volunteer using a spray bandage for carotid artery pulse measurements.

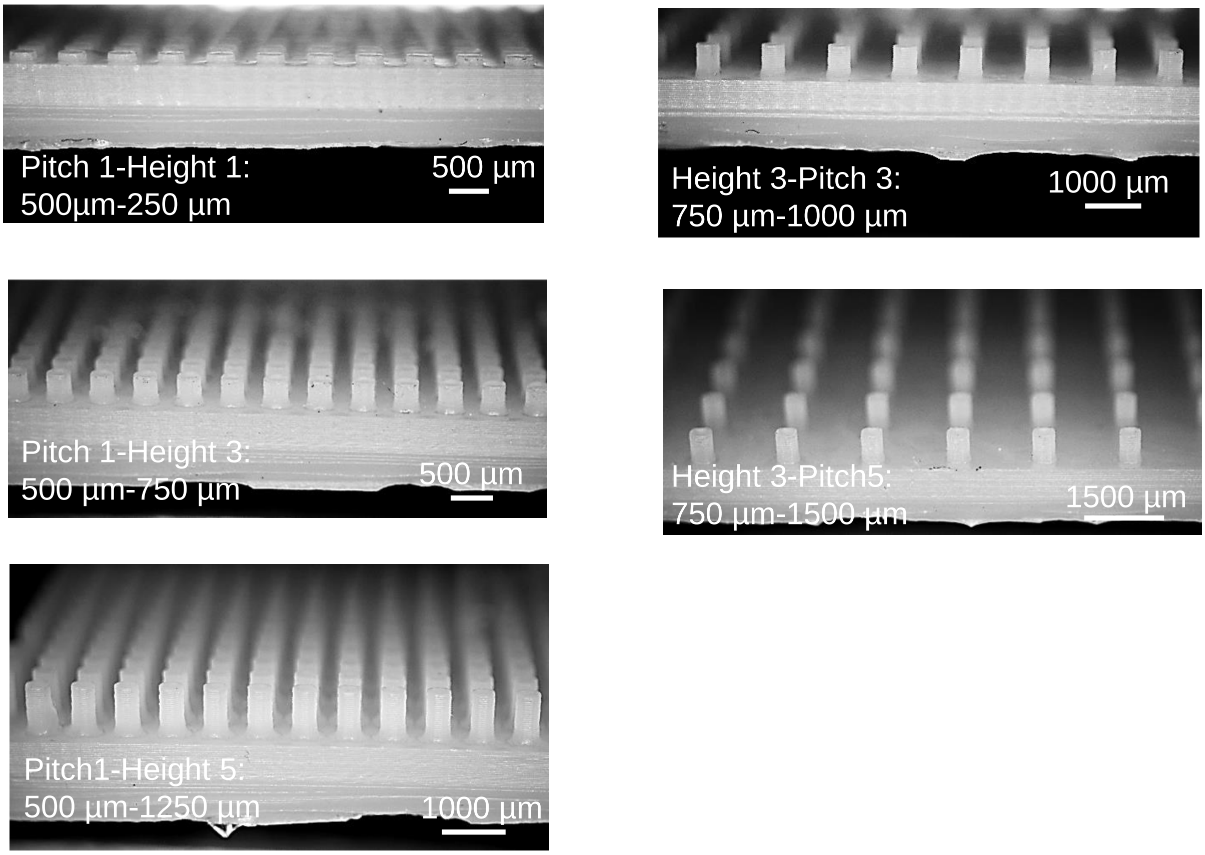

Figure S40. Optical microscopy images of 3D-printed micropillar arrays of various pitch sizes and heights used in the texture perception experiments.

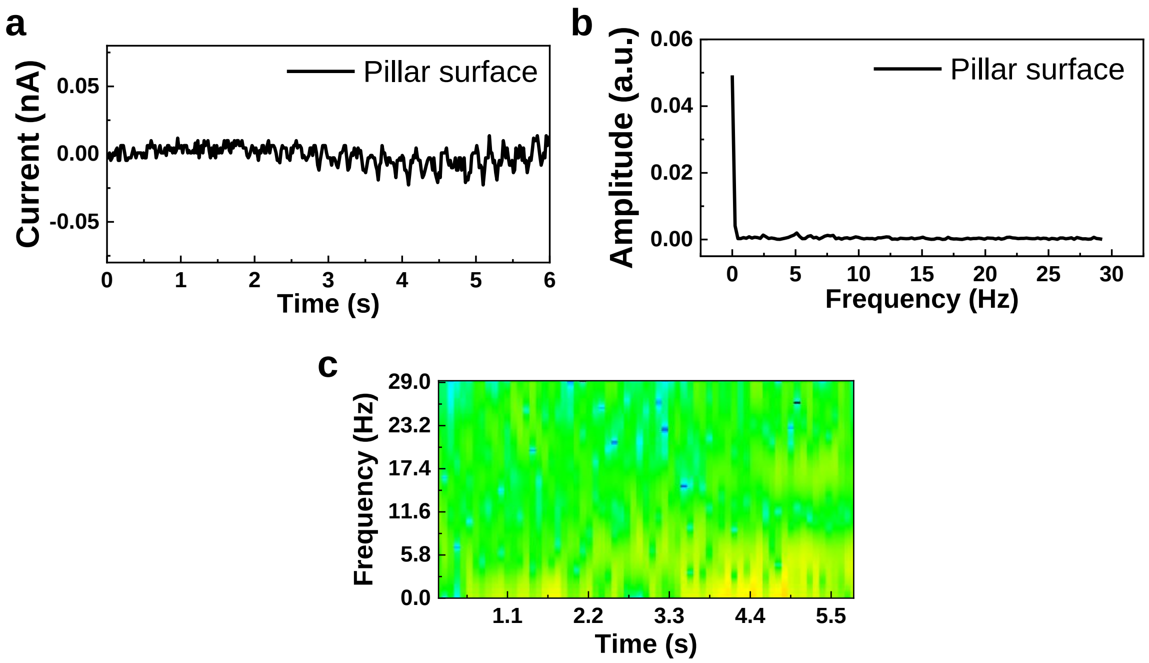

Figure S41. Texture perception results from the planar gelatine film device with a texture amplification layer. (a) The output current from the device showing no distinguishable (b) FFT peak and (c) STFT patterns during sliding over the pillar patterned surface.

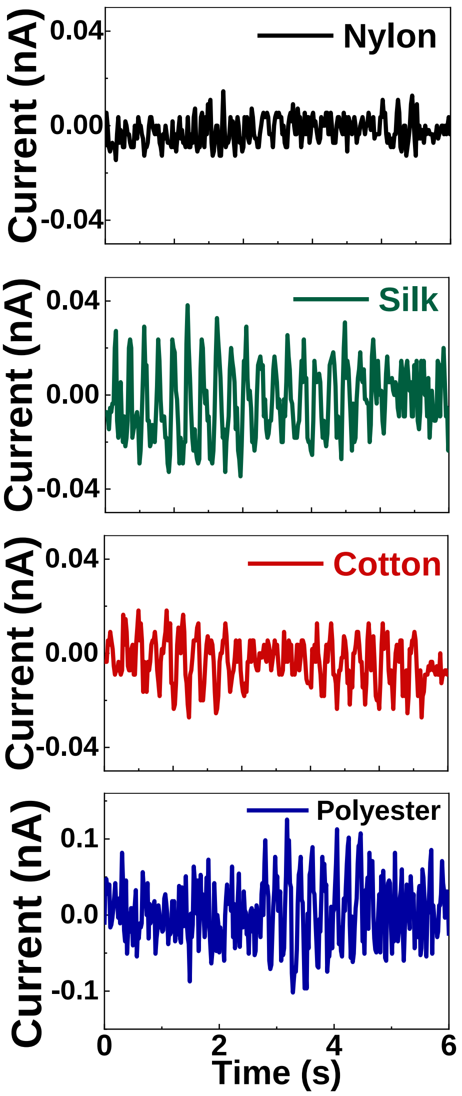

Figure S42. Output current from the interlocked e-skin during texture perception measurements of several commercial textile fabrics.

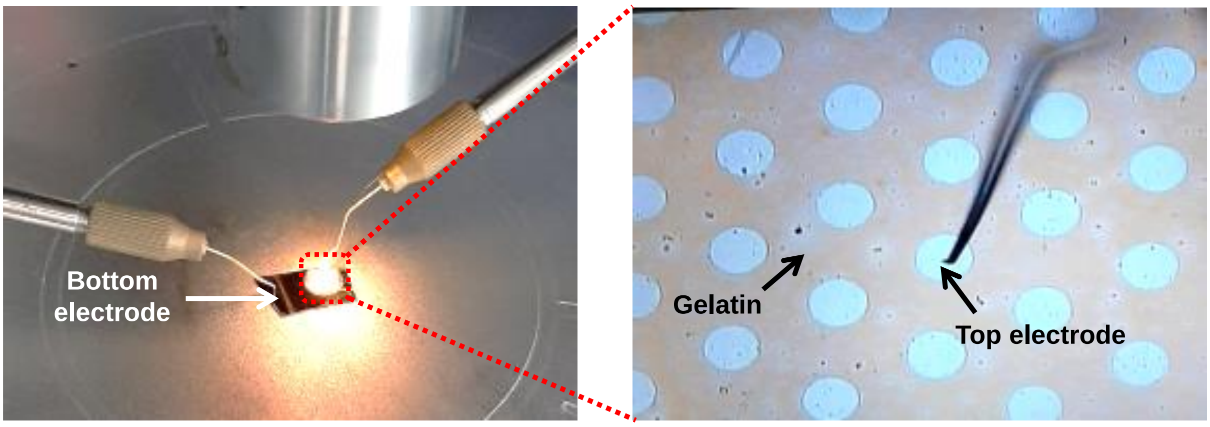

Figure S43. Experimental setup for the hysteresis loop measurements.

Table S1. Cost comparison of conventional piezoelectric biodegradable polymers with gelatin, which is still less explored as a biodegradable pyro/piezo-electric material.

Biopolymer	Specification (Sigma-Aldrich)	Prices[a)] [USD g^{-1}]
**Gelatine (from porcine skin) (Used in this work)**	Gel strength ~300 g Bloom, Type A, BioReagent, for electrophoresis, suitable for cell culture<br>CAS Number 9000-70-8	**34.20/100 = 0.342**
**Cellulose acetate**	Average Mn ~30,000 by GPC<br>CAS Number: 9004-35-7	**51.5/25 = 2.06**
**Collagen (from calf skin)**	Bornstein and Traub Type I, solid, BioReagent, suitable for cell culture<br>CAS Number 9007-34-5	**349/0.25 = 1396**
**Elastin (from bovine neck ligament)**	Powder<br>CAS Number 9007-58-3	**296/10= 2.96**
**Keratin**	MDL number MFCD00131435	**346/5 = 69.2**
**Silk (Fibroin solution, 50 mg/ml)**	Product number: 5154-20ML	**290/1 = 290**
**Chitin (from shrimp shells)**	BioReagent, suitable for analysis of chitinase, purified powder<br>CAS Number 1398-61-4	**379/5 = 75.8**
**Poly(γ- benzyl-L-glutamate)**	mol wt. 70,000-150,000<br>CAS Number 25014-27-1	**649/1 = 649**
**Polylactic Acid (PLA)**	G**ranule, 3 mm nominal granule size, weight 100 g, natural**<br>**SKU-Pack Size: GF45989881-1EA**	**186/100 = 1.86**

[a)]From the website of Sigma-Aldrich (August 2020).

Table S2. Comparison of piezoelectric output performances in terms of the output voltage, current, power, and strain coefficient of previously reported biomaterial-based piezoelectric devices with our developed microdome patterned gelatine device.

Material	Output voltage	Output current	Output power	Piezoelectric coefficient (d_{33})	References
**Microdome patterned interlocked gelatine film**	**1.6 V under 113 kPa (~ 11.3 N)**	**8.9 $nAcm^{-2}$ under 113 kPa**	**0.3 μWcm^{-2} under 113 kPa**	**d_{33} ~ 24 pCN^{-1}**	**This work**
Diphenylalanine (FF) peptide microrod	1.4 V under 60 N	39.2 nA under 60 N	3.3 nW cm^{-2} under 60 N	d_{33} ~ 17.9 pCN^{-1}	2
Diphenylalanine (FF) peptide nanotubes	2.8 V under 42 N	37.4 nA under 42 N	8.2 nW under 42 N	d_{15} ~ 46.6 pCN^{-1}	3
Vertically aligned M13 phage nanofibres	2.8 V under 17 N	120 nA under 17 N	236 nW under 42 N	d_{33} ~ 13.2 pCN^{-1}	4
2D-dot patterns M13 phage	0.95 V under 65 N	94 nA under 65 N	80 nW under 65 N	Not mentioned	5
Cycloglycine-tryptophan (cyclo-GW)	1.2 V under 65 N (18.8 mVN^{-1})	1.75 nA under 65 N	Not mentioned	d_{16}, d_{36} ~ 14 pCN^{-1}	6
γ-glycine	0.45 V under 0.172 N	Not mentioned	Not mentioned	d_{33} ~ 10 pCN^{-1}	7
M13 bacteriophage (phage)	0.4 V under 34 N	4 nA under 34 N	Not mentioned	d_{33} ~ 7.8 pCN^{-1}	8
M13 bacteriophage nanopillars	232 mV under 30 N	11.1 nA under 30 N	0.99 nW under 30 N	d_{33} ~ 10 pCN^{-1}	9
Fish skin based collagen	200 mV under 7.5 N	2.5 nA under 7.5 N	4.6 nW/cm^2 under 7.5 N	d_{33} ~ 3 pCN^{-1}	10

Table S3. Materials parameters of the gelatine films used in the FEM simulations.

Property	Variable	Expression	Unit
**Coupling matrix**	e	{-0.020, 0.004, 0.01, -0.028, 0.011, 0.011, -0.025, -0.009, 0.009, -0.025, -0.006, 0.005, -0.017, 0, -0.033, -0.007, 0.003, -0.006}	C/m^2
**Coupling matrix**	d	{0, 0, 0, 0, 0, 0, -4.84, -4.84, -19.6, -12, 6.21, 0, 6, 12, 0, 0, 0, 0}	pC/N
**Thermal conductivity**	κ	0.21	W/mK
**Specific heat capacity**	C_P	1.2	J/kg.K
**Density**	ρ	1330	kg/m^3
**Poisson's ratio**	ν	0.5	1

Table S4. Comparison of the piezoelectric coefficient of the interlocked gelatine films with previously reported biodegradable, and organic and inorganic non-biodegradable materials.

Type of material	Name of material	Maximum piezoelectric coefficient (pC/N)	References
Biodegradable	Calcite spar	0.02	11
	Wood	0.1	12
	Bone	0.2	13
	L-Threonine (Film)	0.2	14
	Hydroxy-L-Proline (Film)	1.5	
	Elastin	1	15
	Silk	1.5	16
	Keratin (Horn)	1.8	17
	Starch	2	
	Polymethyl-L-Glutamate (PMG)	2	
	Poly-Lactic-Acid (PLA)	10	
	Chitin Nanofibre	2	18
	Hydroxyapatite (Poled Ceramic)	2	19
	Dry Tendon	2	20
	Chitosan	6	21
	Cellulose nanofibrils CNF	8	
	L-Alanine	6	22
	Lysozyme (Film)	6.5	23
	M13 Bacteriophage Virus	7.8	8
	Collagen	12	24
	Glycine Nanofibres	12.5	25
	Gamma Glycine	10	7
	L-Cysteine	11	
	L-Asparagine	13	
	L-Aspartate	13	
	L-Methionine	15	
	L-Histidine	18	
	L-Leucine	20	
	Gelatine Nanofibre	20	26
	**Interlocked gelatine film**	**24**	**This work**
Organic Non-biodegradable	Propylene Oxide	0.1	17
	Poly-β-Hydroxybutyrate	1.3	
	Poly (vinylidene cyanide) (VDCN) and Poly (vinyl acetate) (VAc)	5	
	Polyacrylonitrile	2	27
	Polyamide (Nylon) 11	4	
	Polyimide	2.5−16.5	
	PVDF	24−34	
	P(VDF-HFP)	24	
	P(VDF-TrFE)	25−40	
Inorganic Non-biodegradable	Quartz	2.3	28
	Al_3PO_7	3.4	29
	$GaPO_7$	8.2	
	Al_3AsO_7	9	

	Ga_3AsO_7	23.9	
	$CaBi_4Ti_4O_{15}$ (CBT)	8	30
	AlN	5.1	31
	ZnO Nanorods	9.5	32
	ZnO	9.9	33
	GaN Nanowires	12.8	34
	$BiFeO_3$	18	35

Table S5. The pyroelectric performance comparison of our developed interlocked micro-pattern e-skin over previously reported and state-of-the-art pyroelectric sensors and materials.

Type	Material	Pyroelectric coefficient ($\mu Cm^{-2}K^{-1}$)	Working condition (ΔT, dT/dt)	Output current	References
Biodegradable	Hoof tendon	0.004	-35−85 ℃, 14 ℃min^{-1}	-	36
	Dentine and cementum	0.025 − 0.0015	-	-	37
	Fluorapatite/ gelatine composite	0.05	-	-	38
	Natural human skin	0.021 − 0.27	-	-	39
	dabcoHReO$_4$ fibres	8.5	6 K, 0.2 Ks^{-1}	200 pA	40
	Hydroxyapatite thin film	12	50 ℃, 1 °Cmin^{-1}	4 pA	41
	**Interlocked microdome patterned Gelatine film**	**13**	**1.8 K, 0.4 K/s**	**0.46 nA/cm^2**	**This work**
Organic non-biodegradable	PVDF- graphene oxide (GO) composite nanofibres	0.027	22 K, 2.12 Ks^{-1}	45 pA	42
	PVDF-methylammonium lead iodide ($CH_3NH_3PbI_3$) composite nanofibres	0.044	38 K, 2.26 Ks^{-1}	18.2 pA	43
	PVDF nanofibre	0.062	14 K, 1.5 Ks^{-1}	60 pA	44
Inorganic non-biodegradable	CdSe	3.5	-196−25 °C	-	45
	CdS	4	-196−25 °C	-	
	Gallium nitride (GaN)	4.8	-	-	46
	AlN thin film	6−8	-	-	47
	$KNbO_3$ nanowires	8	39 K, 2 Ks^{-1}	120 pA	48
	Bulk ZnO	9.4	-	-	49
	ZnO thin film	10	2 Kmin^{-1}	0.4 pA	50

Table S6. Pressure sensitivity and detection limit of our developed devices

Device configuration	Voltage sensitivity (mV/Pa)			Current sensitivity (nA/kPa)		Detection limit
	Pressure range			Pressure range		
	< 2 Pa	40-100 Pa	0.1-100 kPa	40 Pa – 1 kPa	1 kPa – 100 kPa	
**Microdome**	41	1	0.026	2.45	0.03	0.005 Pa
**Micropyramid**	9.5	0.47	0.0166	2.26	0.018	0.4 Pa
**Micropillar**	9.3	0.37	0.0069	0.67	0.018	0.4 Pa
**Planar**	5.73	0.35	0.0017	0.018	0.003	0.4 Pa

Table S7. Comparison of the pressure sensitivity and detection limit of our developed microdome patterned e-skin with state-of-the-art pressure sensors

	Material	Sensitivity	Pressure range	Detection limit	Ref.
Biodegradable	Interlocked microdome patterned gelatine film	41 mV/Pa	0.005−1.7 Pa	0.005 Pa	**This work**
		1 mV/Pa	40−100 Pa		
		0.026 mV/Pa	100 Pa−100 kPa		
	Biodegradable β-glycine/chitosan	2.82±0.2 µV/Pa	5−60 kPa		51
	Electrospun gelatine nanofibre	0.8 mV/Pa	< 10 kPa	0.02 Pa	26
	Fish skin based nanogenerator	3.7 µV/Pa	< 300 kPa	0.26 N/10 kPa	10
Organic non-biodegradable	Aligned PVDF-TrFE nanofibres	1.1 mV/Pa	0.1−12 Pa	0.1 Pa	52
	Ferroelectret field-effect transistor	0.1 mV/Pa	0.01−30 Pa	0.01 Pa	53
	P(VDF-TrFE) thin film	0.75 µV/Pa	3.3−13.3 kPa		54
	Vertically integrated P(VDF-TrFE) fibre array	26.94 µV/Pa	40−800 kPa		55
	PVDF/$BaTiO_3$ nanocomposite fibres	17 µV/Pa	1−40 kPa		56
	Eletrospun PVDF fabric	0.83 µV/Pa	150−250 kPa		57
	P(VDF-TrFE) film	0.02 µV/Pa	8.9−444 kPa	11.11 kPa	58
	PVDF-GO nanofibre	4.3 mV/Pa	10−120 Pa	10 Pa	42
	Pt-PVDF aligned nanofibres	1.6 µV/Pa	< 435 kPa	2 N/87 kPa	59
		13.8 µV/Pa	435−870 kPa		
		13.1 µV/Pa	870 kPa−2.17 MPa		
	P(VDF-TrFE)/$BaTiO_3$	2.615 µV/Pa	100−500 kPa	7 kPa	60
Inorganic non-biodegradable	Ultrathin PZT transistor based sensor	0.82 µV/Pa	2−10 Pa	0.005 Pa	61
	vertically aligned ZnO NW	0.019 µV/Pa	20−34 kPa		62

References

1. A. Bot, I. A. van Amerongen, R. D. Groot, N. L. Hoekstra and W. G. Agterof, *Polym. Gels Networks*, 1996, **4**, 189-227.
2. V. Nguyen, R. Zhu, K. Jenkins and R. Yang, *Nat. Commun.*, 2016, *Nat. Commun.* **7**, 13566.
3. J.-H. Lee, K. Heo, K. Schulz-Schönhagen, J. H. Lee, M. S. Desai, H.-E. Jin and S.-W. Lee, *ACS Nano*, 2018, **12**, 8138-8144.
4. J.-H. Lee, J. H. Lee, J. Xiao, M. S. Desai, X. Zhang and S.-W. Lee, *Nano Lett.*, 2019, **19**, 2661-2667.
5. K. Heo, H.-E. Jin, H. Kim, J. H. Lee, E. Wang and S.-W. Lee, *Nano energy*, 2019, **56**, 716-723.
6. K. Tao, W. Hu, B. Xue, D. Chovan, N. Brown, L. J. Shimon, O. Maraba, Y. Cao, S. A. Tofail and D. Thompson, *Adv. Mater.*, 2019, **31**, 1807481.
7. S. Guerin, A. Stapleton, D. Chovan, R. Mouras, M. Gleeson, C. McKeown, M. R. Noor, C. Silien, F. M. Rhen and A. L. Kholkin, *Nat. Mater.*, 2018, **17**, 180-186.
8. B. Y. Lee, J. Zhang, C. Zueger, W.-J. Chung, S. Y. Yoo, E. Wang, J. Meyer, R. Ramesh and S.-W. Lee, *Nat. Nanotechnol.*, 2012, **7**, 351.
9. D.-M. Shin, H. J. Han, W.-G. Kim, E. Kim, C. Kim, S. W. Hong, H. K. Kim, J.-W. Oh and Y.-H. Hwang, *Energy Environ. Sci.*, 2015, **8**, 3198-3203.
10. S. K. Ghosh and D. Mandal, *ACS Sustain. Chem. Eng.*, 2017, **5**, 8836-8843.
11. S. Guerin, S. A. Tofail and D. Thompson, *IEEE Trans. Dielectr. Electr. Insul.*, 2018, **25**, 803-807.
12. E. Fukada, *J. Phys. Soc. Jpn.*, 1955, **10**, 149-154.
13. E. Fukada and I. Yasuda, *J. Phys. Soc. Jpn.*, 1957, **12**, 1158-1162.
14. S. Guerin, S. A. Tofail and D. Thompson, *Cryst. Growth Des.*, 2018, **18**, 4844-4848.

15. Y. Liu, Y. Wang, M.-J. Chow, N. Q. Chen, F. Ma, Y. Zhang and J. Li, *Phys. Rev. Lett.*, 2013, **110**, 168101.
16. T. Yucel, P. Cebe and D. L. Kaplan, *Adv. Funct. Mater.*, 2011, **21**, 779-785.
17. E. Fukada, *IEEE Trans Ultrason. Ferroelectr. Freq. Control.*, 2000, **47**, 1277-1290.
18. S. K. Ghosh and D. Mandal, *Appl. Phys. Lett.*, 2017, **110**, 123701.
19. A. A. Gandhi, M. Wojtas, S. B. Lang, A. L. Kholkin and S. A. Tofail, *J. Am. Ceram. Soc.*, 2014, **97**, 2867-2872.
20. E. Fukada and I. Yasuda, *Jpn. J. Appl. Phys.*, 1964, **3**, 117.
21. A. Hänninen, E. Sarlin, I. Lyyra, T. Salpavaara, M. Kellomäki and S. Tuukkanen, *Carbohydr. Polym.*, 2018, **202**, 418-424.
22. Z. Tylczyński, A. Sterczyńska and M. Wiesner, *J. Condens. Matter Phys.*, 2011, **23**, 355901.
23. A. Stapleton, M. Noor, J. Sweeney, V. Casey, A. Kholkin, C. Silien, A. Gandhi, T. Soulimane and S. Tofail, *Appl. Phys. Lett.*, 2017, **111**, 142902.
24. D. Denning, J. I. Kilpatrick, E. Fukada, N. Zhang, S. Habelitz, A. Fertala, M. D. Gilchrist, Y. Zhang, S. A. Tofail and B. J. Rodriguez, *ACS Biomater. Sci. Eng.*, 2017, **3**, 929-935.
25. D. Isakov, E. d. M. Gomes, I. Bdikin, B. Almeida, M. Belsley, M. Costa, V. Rodrigues and A. Heredia, *Cryst. Growth Des.*, 2011, **11**, 4288-4291.
26. S. K. Ghosh, P. Adhikary, S. Jana, A. Biswas, V. Sencadas, S. D. Gupta, B. Tudu and D. Mandal, *Nano Energy*, 2017, **36**, 166-175.
27. S. Mishra, L. Unnikrishnan, S. K. Nayak and S. Mohanty, *Macromol Mater Eng*, 2019, **304**, 1800463.
28. R. Bechmann, *Phys. Rev.*, 1958, **110**, 1060.
29. J. Ren, Z. Ma, C. He, R. Sa, Q. Li and K. Wu, *Comput. Mater. Sci.*, 2015, **106**, 1-4.

30. D. Peng, X. Wang, C. Xu, X. Yao, J. Lin and T. Sun, *J. Am. Ceram. Soc.*, 2013, **96**, 184-190.

31. C. Lueng, H. L. Chan, C. Surya and C. Choy, *J. Appl. Phys.*, 2000, **88**, 5360-5363.

32. D. A. Scrymgeour and J. W. Hsu, *Nano Lett.*, 2008, **8**, 2204-2209.

33. I. Kobiakov, *Solid State Commun.*, 1980, **35**, 305-310.

34. M. Minary-Jolandan, R. A. Bernal, I. Kuljanishvili, V. Parpoil and H. D. Espinosa, *Nano Lett.*, 2012, **12**, 970-976.

35. M. Graf, M. Sepliarsky, R. Machado and M. G. Stachiotti, *Solid State Commun.*, 2015, **218**, 10-13.

36. S. B. Lang, *Nature*, 1966, **212**, 704-705.

37. H. Athenstaedt, *Arch. Oral Biol.*, 1971, **16**, 495-501.

38. M. Burgener, T. Putzeys, M. P. Gashti, S. Busch, H. Aboulfadl, M. Wübbenhorst, R. d. Kniep and J. r. Hulliger, *Biomacromolecules*, 2015, **16**, 2814-2819.

39. H. Athenstaedt, H. Claussen and D. Schaper, *Science*, 1982, **216**, 1018-1020.

40. D. Isakov, E. de Matos Gomes, B. Almeida, A. Kholkin, P. Zelenovskiy, M. Neradovskiy and V. Y. Shur, *Appl. Phys. Lett.*, 2014, **104**, 032907.

41. S. Lang, S. Tofail, A. Gandhi, M. Gregor, C. Wolf-Brandstetter, J. Kost, S. Bauer and M. Krause, *Appl. Phys. Lett.*, 2011, **98**, 123703.

42. K. Roy, S. K. Ghosh, A. Sultana, S. Garain, M. Xie, C. R. Bowen, K. Henkel, D. Schmeiβer and D. Mandal, *ACS Appl. Nano Mater.*, 2019, **2**, 2013-2025.

43. A. Sultana, S. K. Ghosh, M. M. Alam, P. Sadhukhan, K. Roy, M. Xie, C. R. Bowen, S. Sarkar, S. Das and T. R. Middya, *ACS Appl. Mater. Interfaces*, 2019, **11**, 27279-27287.

44. S. K. Ghosh, M. Xie, C. R. Bowen and D. Mandal, *AIP Conf. Proc.* 2018, **1942**, 140025.

45. D. Berlincourt, H. Jaffe and L. Shiozawa, *Phys. Rev.*, 1963, **129**, 1009.

46. Y.-R. Wu and J. Singh, *J. Appl. Phys.*, 2007, **101**, 113712.

47. V. Fuflyigin, E. Salley, A. Osinsky and P. Norris, *Appl. Phys. Lett.*, 2000, **77**, 3075-3077.
48. Y. Yang, J. H. Jung, B. K. Yun, F. Zhang, K. C. Pradel, W. Guo and Z. L. Wang, *Adv. Mater.*, 2012, **24**, 5357-5362.
49. S. B. Lang, *Phys. Today*, 2005, **58**, 31.
50. C. p. Ye, T. Tamagawa and D. Polla, *J. Appl. Phys.*, 1991, **70**, 5538-5543.
51. E. S. Hosseini, L. Manjakkal, D. Shakthivel and R. Dahiya, *ACS Appl. Mater. Interfaces*, 2020, **12**, 9008-9016.
52. L. Persano, C. Dagdeviren, Y. Su, Y. Zhang, S. Girardo, D. Pisignano, Y. Huang and J. A. Rogers, *Nat. Commun.*, 2013, **4**, 1-10.
53. I. Graz, M. Kaltenbrunner, C. Keplinger, R. Schwödiauer, S. Bauer, S. P. Lacour and S. Wagner, *Appl. Phys. Lett.*, 2006, **89**, 073501.
54. T. Sharma, S.-S. Je, B. Gill and J. X. Zhang, *Sens. Actuator A Phys.*, 2012, **177**, 87-92.
55. X. Chen, H. Tian, X. Li, J. Shao, Y. Ding, N. An and Y. Zhou, *Nanoscale*, 2015, **7**, 11536-11544.
56. W. Guo, C. Tan, K. Shi, J. Li, X.-X. Wang, B. Sun, X. Huang, Y.-Z. Long and P. Jiang, *Nanoscale*, 2018, **10**, 17751-17760.
57. Y. Wang, J. Zheng, G. Ren, P. Zhang and C. Xu, *Smart Mater. Struct.*, 2011, **20**, 045009.
58. C. Li, P.-M. Wu, S. Lee, A. Gorton, M. J. Schulz and C. H. Ahn, *J Microelectromech. Syst.*, 2008, **17**, 334-341.
59. S. K. Ghosh and D. Mandal, *Nano Energy*, 2018, **53**, 245-257.
60. N. T. Tien, S. Jeon, D. I. Kim, T. Q. Trung, M. Jang, B. U. Hwang, K. E. Byun, J. Bae, E. Lee and J. B. H. Tok, *Adv. Mater.*, 2014, **26**, 796-804.
61. C. Dagdeviren, Y. Su, P. Joe, R. Yona, Y. Liu, Y.-S. Kim, Y. Huang, A. R. Damadoran, J. Xia and L. W. Martin, *Nat. Commun.*, 2014, **5**, 1-10.

62. Y. Zhao, Y. Fu, P. Wang, L. Xing and X. Xue, *Nanoscale*, 2015, **7**, 1904-1911.
